# Bi-Directional Tuning of Amygdala Sensitivity in Combat Veterans Investigated with fMRI

**DOI:** 10.1371/journal.pone.0130246

**Published:** 2015-06-29

**Authors:** Tom Brashers-Krug, Ricardo Jorge

**Affiliations:** 1 Department of Psychiatry, University of Iowa Carver College of Medicine, Iowa City, Iowa, United States of America; 2 Department of Psychiatry, Iowa City VA Healthcare System, Iowa City, Iowa, United States of America; 3 Mental Health Service Line, Veterans Affairs Medical Center, Houston, Texas, United States of America; 4 Department of Psychiatry and Behavioral Sciences, Baylor College of Medicine, Houston, Texas, United States of America; West China Hospital of Sichuan University, CHINA

## Abstract

**Objectives:**

Combat stress can be followed by persistent emotional consequences. It is thought that these emotional consequences are caused in part by increased amygdala reactivity. It is also thought that amygdala hyper-reactivity results from decreased inhibition from portions of the anterior cingulate cortex (ACC) in which activity is negatively correlated with activity in the amygdala. However, experimental support for these proposals has been inconsistent.

**Methods:**

We showed movies of combat and civilian scenes during a functional magnetic resonance imaging (fMRI) session to 50 veterans of recent combat. We collected skin conductance responses (SCRs) as measures of emotional arousal. We examined the relation of blood oxygenation-level dependent (BOLD) signal in the amygdala and ACC to symptom measures and to SCRs.

**Results:**

Emotional arousal, as measured with SCR, was greater during the combat movie than during the civilian movie and did not depend on symptom severity. As expected, amygdala signal during the less-arousing movie increased with increasing symptom severity. Surprisingly, during the more-arousing movie amygdala signal *decreased* with increasing symptom severity. These differences led to the unexpected result that amygdala signal in highly symptomatic subjects was lower during the more-arousing movie than during the less-arousing movie. Also unexpectedly, we found no significant inverse correlation between any portions of the amygdala and ACC. Rather, signal throughout more than 80% of the ACC showed a strong positive correlation with signal throughout more than 90% of the amygdala.

**Conclusions:**

Amygdala reactivity can be tuned bi-directionally, either up or down, in the same person depending on the stimulus and the degree of post-traumatic symptoms. The exclusively positive correlations in BOLD activity between the amygdala and ACC contrast with findings that have been cited as evidence for inhibitory control of the amygdala by the ACC. The conceptualization of post-traumatic changes in neural function should be reconsidered.

## Introduction

The emotional effects of prolonged combat have been documented as far back as Homer’s description of Achilles’ behavior during the battle of Troy [1]. They include autonomic hyper-arousal, excessive vigilance, social withdrawal, and sudden anger. These symptoms can remain even after soldiers leave combat behind and return to civilian life. Although some veterans experience symptoms extreme enough both in intensity and duration to merit consideration as a distinct mental syndrome such as PTSD, these symptoms present along a continuum. To emphasize the continuous nature of these phenomena, we will often avoid the term “PTSD” and refer instead to “symptoms of post-traumatic emotional distress.”

It is widely believed that alterations in the neural circuitry underlying fear processing and threat assessment are responsible, at least in part, for many post-traumatic symptoms [2–4]. Although this does not imply that fear is itself a primary cause of post-traumatic emotional distress, there has been a persistent historical overlap between research into the neural mechanisms of fear and those of PTSD. For example, as far back as 1954, it was proposed that fear conditioning elicited by exposure to extremely intense stressful stimuli may be difficult to reverse, and speculations were made that the cingulate cortex and amygdala were among the neural structures involved in these partially irreversible fear responses [5]. Since that time, the components and dynamics of this neural circuitry have been investigated in a vast body of work both in humans and in animals. Investigations in animals have employed lesion methods [6], electrical stimulation [7], and pharmacologic [8] and optogenetic manipulation [9]. In humans, work has included psychophysical experiments on measures of arousal [10,11], functional neuroimaging studies [3,12,13], and even naturalistic lesion studies [14].

In this report, we will point out two basic conceptualizations that have emerged, limiting our discussion to the amygdala and ACC (or the likely homologue of the ACC in rodents, the medial prefrontal cortex (mPFC)).

Activity in the amygdala is thought to play a role in identifying danger [15,16], uncertainty [17], and other behaviorally relevant stimuli in the environment [18]. The exact nature of this role is still a matter of investigation and debate, but it is likely that the amygdala directs attention [19], increases arousal [20], enhances memory for emotionally salient stimuli [21], and that it is required for aversive classical conditioning [22–24],

The ACC has reciprocal connections with portions of the amygdala [25]. Experiments in rodents have demonstrated that stimulating cells in some regions of the mPFC can suppress amygdala activity [7] and consequently decrease fear-related conditioning and behavior [16]. The rodent mPFC can be divided into infra-limbic (IL) and pre-limbic (PL) regions. Some evidence suggests that although amygdala activity may be *inhibited* by the IL it may be *excited* by the PL [26,27]. It is possible that the human sub-genual ACC (also known as BA25) corresponds to IL in the rodent, and human rostral or dorsal ACC (rACC or dACC) corresponds to PL, but such homologies are far from exact [28]. In any event, research on PTSD has tended to focus on the inhibitory effect of the mPFC and ACC on the amygdala [29].

Using this data and informing the data’s interpretation, a consensus model has emerged. This model is not always described in its entirety and appears most often in reviews rather than in original research articles. Nor are all researchers and clinicians in complete agreement with this model. It remains nevertheless the most widely cited and discussed formulation and can therefore reasonably be referred to as the standard neurocircuitry model of PTSD (or, hereafter, simply the “standard model”).

In the standard model, many symptoms of post-traumatic emotional distress are in large part the result of increased amygdala reactivity [3,30,31]. The increase in amygdala reactivity in turn results from decreased inhibitory drive from the ACC.

### The Standard Model: Amygdala

Increases in amygdala reactivity to emotionally provocative stimuli have been reported whether or not the stimuli were related to prior stressful events [30–32]. This has been seen as evidence that symptomatic subjects demonstrate impaired extinction learning [11] and that they fail at some level to differentiate between those cues, environments, and situations that are associated with safety and those that are not [33].

### The Standard Model: ACC

Influential early neuroimaging studies of patients with PTSD found evidence of a negative correlation between neural activity in the ACC and neural activity in the amygdala [3,34]. That is, *increased* amygdala activity was found to coincide with *decreased* ACC activity, and *decreased* amygdala activity was found to coincide with *increased* ACC activity [32,35]. This has led to the proposal that the amygdala hyper-reactivity sometimes seen in PTSD results from decreased inhibitory input from a hypo-active ACC [36]. Studies in non-clinical populations also have suggested that portions of the ACC may exert a “top-down”, inhibitory control over the amygdala [37–39], perhaps analogous to the findings in animal studies cited above [7,16]. Although it remains to be seen whether the ACC directly inhibits the amygdala in humans, animal studies, primarily in rodents, suggest a neural circuitry in which this is possible [7,40,41].

### The Standard Model: Shortcomings

The recent advent of targeted neurostimulation and finely directed neurosurgery as treatment modalities for brain-based disorders [42,43] demands accurate assessment of network pathology. Despite its general acceptance as a theoretical framework, there are limitations to the standard model. A few studies have reported findings that contradict the standard model outright. One study [44] and one meta-analysis [29] have reported functional brain imaging evidence for decreased amygdala activation in subjects with PTSD. Another study reported results from brain-injured combat veterans suggesting that decreased activation in the ACC can protect against development of PTSD [14], a result opposite to that predicted by the standard model. A more sizable number have failed to confirm the model’s predictions about increased amygdala responsiveness [45–48] and the negative correlation between the amygdala and ACC [49–51]. Although these negative results may be due in part to the challenges inherent in studying amygdala function in humans, it has not been clear to what extent they represent Type II statistical error or a limitation of the model itself.

One of the challenges in studying the human amygdala is the fact that it habituates to emotional stimuli fairly rapidly, often within a fraction of the time involved in a typical brain-imaging run of several minutes [52,53]. This study was motivated by our desire to develop and test a method by which the amygdala can be activated while avoiding habituation. We predicted that such a method would allow a more robust means to test the interconnections between the amygdala and the ACC. We made use of the fact that “real-world” stimuli such as movies can elicit robust, prolonged patterns of neural activity that are consistent across subjects [54,55]. Movies, therefore, offered the potential of inducing a neural response with less habituation and with more inter-subject reliability.

In accordance with the standard model, we hypothesized that 1) the amygdala’s response to both movies would be higher in proportion to symptoms of post-combat emotional distress; 2) that the amygdala’s response to the combat movie would be higher than that to the civilian movie (see Discussion section for an explanation of why we can not attribute any difference in response solely to the fact that one movie showed combat and the other did not); and, 3) that we would find a negative correlation between BOLD signal in the amygdala and BOLD signal in at least some portions of the ACC.

Compared to unaffected veterans, veterans with symptoms of post-combat distress can experience a diverse range of stimuli as being more highly aversive. To our knowledge, there has not been a comparison of responses to differing levels of emotionally aversive stimuli. Therefore, although we predicted that subjects would have increased amygdala BOLD signal as a function of symptom severity during both movies, and that all subjects would show greater amygdala BOLD signal during the combat movie than during the civilian movie, we hoped to learn from this study whether the function relating increased BOLD signal to symptom severity would be identical for both movies **(S1 Fig)**.

## Materials and Methods

### Participants

Subjects were 50 US veterans (48 males) who had been deployed to either Iraq or Afghanistan within the prior 10 years. Results were not altered when the two female subjects were excluded from the analysis **(S1 Table)**. Subjects were recruited from consecutive referrals to the Polytrauma Services at the Iowa City Veterans Affairs Medical Center. Exclusion criteria were penetrating head injuries, evidence of spinal cord injury, evidence of a neurological condition other than mild traumatic brain injury (mTBI), neuro-immunological disorder, schizophrenia, schizoaffective disorder, or severe co-existing medical illnesses. mTBI, which was not an exclusion criterion, was assessed based on participants’ retrospective reports using the Mayo Clinic Classification Guidelines [56]. Subjects were still eligible to enroll in the study if they had been diagnosed with other psychiatric disorders, if they were taking psychiatric medications, or if they had a history of drug or alcohol abuse. Subjects were not required to stop taking medications prior to scanning [57]; this and other potential confounds were included in post-hoc analyses and did not alter the significant findings **(S2 Table)**. The protocol was approved by the Institutional Review Board for the Iowa City VA Medical Center and for the University of Iowa Hospitals and Clinics. All participants provided written informed consent after the purpose and nature of the study was explained to them. Participants received financial reimbursement.

### Assessments

Psychiatric assessment included the Mini-International Neuropsychiatric Interview (MINI) [58], the Clinician-Administered PTSD Scale (CAPS) [59], the Hamilton Depression rating Scale (HDRS) [60], the Deployment Risk and Resilience Inventory (DRRI) [61], and the AUDIT [62]. The severity of post-combat emotional distress was assessed by means of the CAPS, which yields a score that can range from 0 to 120. CAPS score was treated as a continuous variable. Combat experience and lifetime trauma exposure were each assessed with the DRRI.

Our planned analyses used CAPS scores as the predictor variable for each of the fMRI measurements described below. We used CAPS scores because they provide a reliable measure of the symptoms of post-traumatic emotional distress, and we were interested in examining how such symptoms relate to fMRI measures of brain function. To see whether additional factors might also contribute to the variance of the fMRI data, we ran each of the analyses with every possible combination of the variables in Table 1. We then selected the combination that resulted in the best-fit model (by Akaike Information Criterion (AIC)) for that fMRI measurement. These models did not alter the pattern of significance of our results **(S2 Table)**.

**Table 1:** Demographic characteristics.

Characteristic		
	N	%
Male	48	96%
Number of deployments		
1	21	42%
2	23	46%
3 or more	6	12%
Major Depressive Disorder Diagnosis	29	58%
Alcohol Use Disorder	29	58%
Substance Use Disorder	3	6%
Psychotropic medication use	32	64%
mTBI using Mayo Classification ref		
None	12	24%
Possible	26	52%
Probable	12	24%
	Mean	SD
Age in years	30.8	7.3
Clinician Administered PTSD Scale score	55.6	30.4
Months since TBI or since return from most recent deployment in those without TBI	53.5	22.5

### Behavioral Task

Subjects were shown two movies during a 5-minute run of fMRI scanning. Both movies were downloaded from YouTube. Movies included the continuous audio track of the sounds recorded during filming. One movie was combat-related, filmed from a camera attached to a soldier’s helmet during foot patrol in the mountains of Afghanistan **(S1 Movie)**. The other was a civilian scene, filmed through the front windshield of a car driving south on Lake Shore Drive into downtown Chicago **(S2 Movie)**. The civilian movie was shown first and the combat second to 26 subjects. The order of presentation, which was determined pseudo-randomly, was reversed for the other 24 subjects. Although movies have the advantage that they resemble real-world stimuli, they have the disadvantage that long-duration, complex stimuli are very difficult to match in terms of their low-level perceptual features. Attributes such as motion, luminance, visual contrast, and overall power across auditory and visual spectral frequencies vary constantly throughout each movie and across movies. We will touch on the attendant benefits and disadvantages of using movies as stimuli in the Discussion.

### fMRI Run

**Fig 1:**
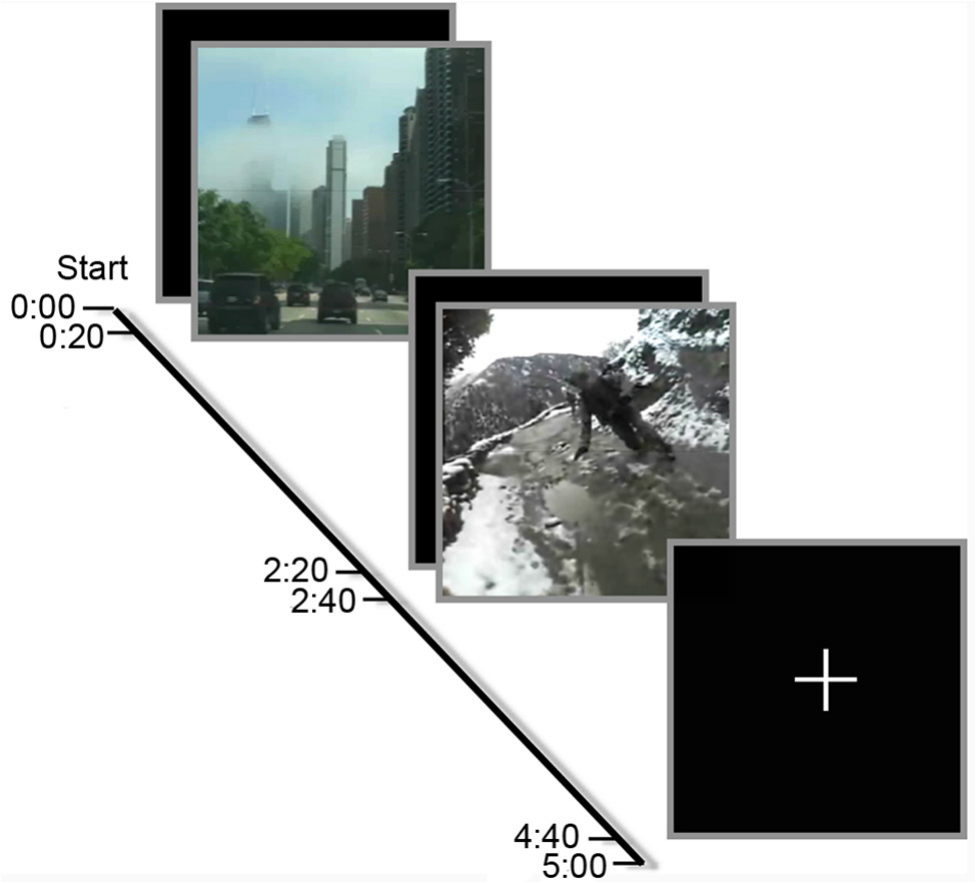
Graphic depiction of the stimulus presentation sequence. Subjects saw two 2-minute movies with each movie preceded and followed by a 20-second white fixation cross centered on a black background.

Each run consisted of an initial 20-second white cross in the center of a black background (fixation), followed by the first 2-minute movie, a second 20-second fixation, the second 2-minute movie, and finally a third 20-second fixation (Fig 1). Movies and the fixation cross were back-projected onto a translucent screen that was viewed via mirrors attached to the fMRI head coil.

### Participant Instructions

Participants wore Avotec Silent Scan headphones. They were instructed to lie still in the scanner, to watch each movie in its entirety, and to keep their eyes open, aside from normal blinking, during the fixation periods as well. We monitored the gaze only of the final 23 participants with an Avotec RE-5701 Eye Tracker. It was only after the first 27 had been scanned that this option was available to us. All 23 of these subjects kept their eyes open during the entire acquisition of fMRI data without the need for reminder or correction by staff. Although we did not monitor subjects’ gaze for the initial 27 subjects, the fact that we did not need to intervene for any of the final 23 suggests that the initial 27 subjects kept their eyes open during the procedure as well. Additionally, we analyzed data on the first 27 subjects (gaze not monitored) and the last 23 subjects (gaze monitored) separately. The results were similar in each subset to those of the entire group. Results from each subset often approached and, in the majority of cases reached, statistical significance **(S3 Table)**.

We wanted the participants to watch the movies without consciously monitoring their subjective experience as they were watching. Because of this, we did not ask them immediately after the first movie how they had felt while watching it. When we initially designed our paradigm, we asked after the second movie how subjects had felt during each movie, and we found that participants could not reliably recall what their subjective state had been during first movie, especially when the combat movie came second. Therefore, we did not collect data on participants’ subjective emotional reactions, sensations, or flashbacks as they watched the movies. Instead, we used SCRs to index their level of arousal, as these have been demonstrated to be reliable indicators of emotional arousal in subjects with PTSD [63].

### Image Acquisition

Participants lay supine in the 3T Siemens TIM Trio MRI Scanner with a 12-channel head coil. Head movement was restricted using foam padding. Functional scans: T2* weighted BOLD images (gradient echo EPI, TE/TR: 30/2000 ms, flip angle: 70°, FOV: 220*220 mm, matrix: 64*64, 5 mm thickness, no gap, 30 slices, interleaved acquisition). The first four scans, along with an initial dummy scan, were discarded to allow for T1 equilibration.

### SCR Recording

Skin conductance was recorded throughout the fMRI session using a BIOPAC MP150 data acquisition system (BioPac Systems). Disposable silver/silver-chloride electrodes (BioPac EL507) with isotonic 0.5% sodium chloride conductance gel were placed on the sole of the left foot. The area of skin on the foot was first cleaned with an alcohol swab and allowed to air dry before electrode placement. To quantify SCRs, we followed the method proposed in Boucsein [64] for finding the area under an SCR as detailed in the appendix of that book. In brief, we first located and recorded the maximum amplitude at the peak of an SCR. We then located the point before and the point after the peak where the value equaled half the maximum value (half-max) of the peak. The time that elapsed between the two half-max points was taken as the duration of the SCR. Finally, the area of the SCR was defined as the product of the maximum value and the duration **(S2 Fig)**. For statistical analysis, SCR areas were the incremented by one and log_10_ transformed to improve normality of the distribution. Henceforth, when we refer to the value of an SCR, we will be referring to the area of that SCR calculated and transformed as just described.

### Functional MRI pre-processing and data analysis

T2* weighted BOLD images (henceforth referred to as “images”) were pre-processed using SPM8 as follows. First, images were slice-time corrected to the initially acquired slice of each image. Next, motion correction was performed on each subjects’ images using a two-step rigid-body transformation. Images were next normalized to a standard stereotactic space (MNI152) using the SPM8 EPI template, resampled into 3 mm isotropic voxels, and smoothed with an 8 mm FWMH Gaussian kernel. We then used Rex (Region-of-interest Extraction toolbox; gablab@mit.edu) to extract voxel-level time courses based on region of interest (ROI) masks created with WFU PickAtlas’s [65,66] automated anatomic labeling (aal) [67]. The PickAtlas’s masks sampled the data at a resolution of 2 mm^3^. We included Brodmann Area BA25, which is sometimes called subgenual ACC, in our ACC ROI mask. After extraction, each voxel’s time course was standardized (zero mean and unit sample variance). Once voxel-level time courses were extracted, all subsequent analyses were done in Mathematica, using custom scripts written by author TBK.

#### Variables of interest

1) Mean amygdala signal. We conducted our activation-based analysis on the bilateral amygdala to minimize the number of comparisons in our statistical tests. Signal in the right and left amygdala were highly correlated with each other (S1 Text). We first identified the points in the signal time courses that corresponded to the first movie, the second movie, and the fixation periods preceding each movie (S2 Text). To determine the mean amygdala signal during a movie, we averaged the signal of all voxels in the amygdala bilaterally during that movie, resulting in a single number. Next, we adjusted this value to take into account the level of signal preceding the movie by subtracting the average of these same voxels during the preceding fixation period. We will refer to the resulting value as the “mean amygdala signal.” This method is conceptually and mathematically similar to the traditional “boxcar regression” method, but provides several advantages for studies in which *a priori* models of the expected time course of BOLD signal are not necessarily applicable (S3 Text). We will mention one important difference here, using terminology as it is used in SPM: because it does not involve fitting data to a model, use of each subject’s mean signal does not require mixed-effects models to generalize results beyond the specific subjects studied.

2) Correlations between amygdala and ACC. To avoid potential bias in voxel selection and to gain both a coarse- and a fine-grained description, this was done on two spatial scales: that of the entire bilateral ROI and that of the individual voxel. For both ROI-level and voxel-level analyses, we included signal from the entire fMRI run to calculate correlations. On the level of the ROI, we averaged each subject’s signal time course over all voxels for each ROI bilaterally. We then calculated the Pearson’s correlation between the average amygdala and the average ACC time courses for each subject. On the level of individual voxels, rather than averaging the time courses across all voxels in each ROI, we retained each voxel’s individual time course. In describing our methods here and in the Supplementary Information, we will use the notation “amy_1_::acc_1_” to indicate a correlation between the time courses of the first amygdala voxel, amy_1_, and the first ACC voxel, acc_1_. (The numbering of the voxels is arbitrary and has no effect on the results, but does facilitate concise descriptions of our methodology.) With the masks’ voxel dimensions of 2 x 2 x 2 mm, there were 248 voxels in the right amygdala and 220 in the left amygdala. Including BA25 in our ACC masks yielded 1500 voxels in the right ACC and 1604 in the left ACC. Thus, for each subject, we obtained a total of 724,880 correlation coefficients – 372,000 (248 x 1500) on the right and 352,880 (220 x 1604) on the left. We calculated the Pearson’s correlation coefficient for every right-sided amy_m_::acc_n_ pair and every left-sided amy_m_::acc_n_ pair separately. We then calculated the single-sample, two-tailed *t*-score of the correlation coefficient for each amy_m_::acc_n_ voxel pair across all subjects.

#### Regressors and nuisance variables

In our activation-based analysis of mean amygdala signal during each movie, we used BOLD signal as described above without modeling potential nuisance regressors (for explanation and justification, see S4 Text). In our correlation analysis, we fit each voxel’s signal time course to the mean signal from eroded masks of the cerebrospinal fluid and cerebral white matter and retained the residuals. To account for potential movement-related artifact, we followed the work of Calhoun, et al. [68] and used impulse functions to remove the effect of individual time points in which a measure of a subject’s motion (Mean Squared Difference (MSD)) exceeded one standard deviation of the mean of all subjects’ MSD.

#### Statistical significance in the face of multiple correlations

Choosing a threshold for statistical significance for 724,880 comparisons poses a challenge. We decided to make use of recent advances in computing power that allow for the very large numbers of calculations required to perform permutation-based simulations. We adapted a three-step approach that has been used previously to analyze inter-subject correlations in fMRI data generated during movie-viewing tasks [54,55]. Step 1: Define a null-distribution: We calculated Pearson’s amy_m_::acc_n_ correlation coefficients in 10,000 iterations of randomized sign-permutations of voxel time courses to establish a null distribution for each voxel pair (S3 Fig). Step 2: Estimate the relevant statistics of the null distribution: For each iteration, we calculated single-sample, two-tailed *t*-scores on the correlation coefficients for each amy_m_::acc_n_ voxel pair across all subjects. This yielded a final total of 10,000 *t*-matrices for the null distribution (S4 Fig). Step 3: Compare statistics of real data to that of the null-distribution data: We were then able to assign each amy_m_::acc_n_ *t*-score in our actual data a *z*-score based on the 10,000 null-distribution *t*-matrices (S5 Fig). We set the strict criterion *z*-score (two-tailed) for each voxel at ± 5.39 (alpha = 0.025/724,880) to correct for the large number of comparisons. This strict criteria allowed us to analyze the anatomic localization with greater spatial precision than cluster-based methods allow.

In a separate and final analysis, we examined the relationship between the voxel-level correlations and symptoms of post-traumatic emotional distress. We calculated the Pearson’s correlation coefficient across participants between each amy_m_::acc_n_ and CAPS scores. In order to define the null distribution, we performed 10,000 iterations in each of which we randomly assigned a CAPS score to each participant (using random selection with replacement). For each iteration, we calculated the Pearson’s correlation coefficient across participants between each amy_m_::acc_n_ and the randomly assigned CAPS scores. In this way, we were able to simulate a population in which a participant’s amy_m_::acc_n_ voxel-level correlations were not related to that participant’s CAPS score **(S5 Text)**.

## Results

Demographic characteristics and results of neuropsychiatric questionnaires were presented in **Table 1**.

### SCR

Data from 43 subjects was retained for the SCR analyses. We were unable to use SCR data from 7 subjects. In one subject, the recording electrodes fell off 30 seconds into the fMRI run. In the other 6, the recorded signal contained no SCRs during the entire fMRI run and consisted of a high degree of noise.

As expected, SCRs were greater during the combat movie than during the civilian movie [paired t-test, *t(42)* = -4.31, *p* = 0.0001]. There was no correlation between CAPS scores and SCRs during either the civilian movie [Pearson’s *r* = -0.19, *p* = 0.22] or the combat movie [Spearman’s *ρ* = -0.13, *p* = 0.432], or between CAPS scores and the difference in SCRs during the two movies [Pearson’s *r* = 0.05, *p* = 0.98]. There was also no correlation between any of the three CAPS sub-scales and SCRs **(S4 Table)**.

Because of the complex nature of the stimuli, we can not be certain which characteristics of the two movies led to the differences in arousal (see **Discussion**). In order to avoid any implication that the results reported here can be attributed solely to the combat or civilian setting of the two movies, we will refer to the movies as “more-arousing” and “less-arousing” for the combat and civilian movie, respectively.

### Mean Amygdala Signal

**Fig 2:**
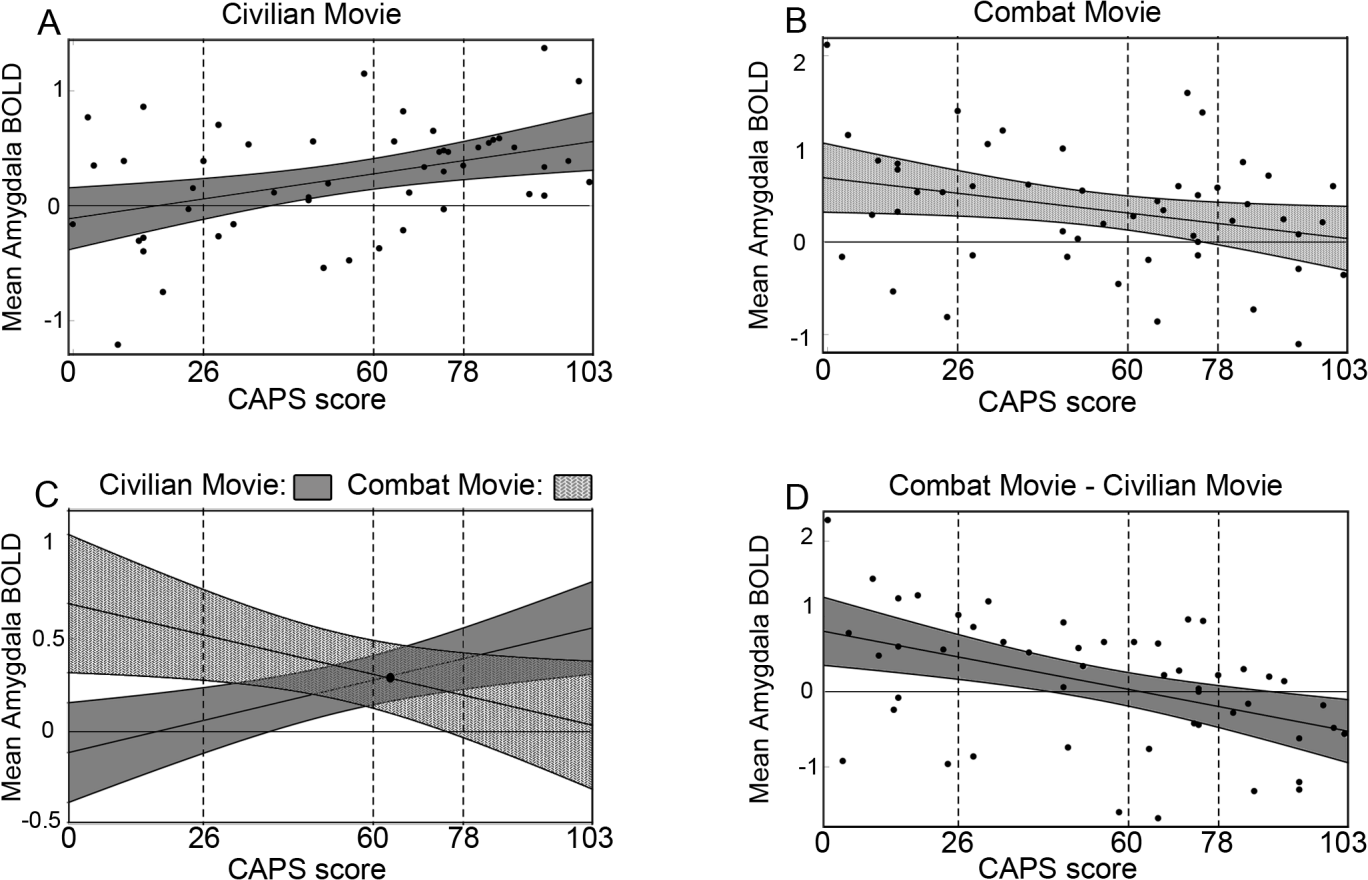
Plots of mean amygdala signal versus CAPS scores. Mean BOLD signal was calculated as described in Methods, and is displayed in arbitrary units. **A)** Mean amygdala signal during the less-arousing movie plotted against CAPS scores (black dots; *r* = 0.40, *p* = 0.0041); also shown are predicted mean amygdala signal (black line) as a linear function of CAPS scores and 95% prediction confidence intervals (CI) depicted by gray shaded region. Quartiles of CAPS scores are indicated by the three vertical dashed lines. **B)** Mean amygdala signal, predicted mean amygdala signal, and 95% prediction CI as a function of CAPS for the more-arousing movie (*r* = -0.29, *p* = 0.039), plotted as in Fig 2A. **C)** Predicted means and 95% CI as functions of CAPS scores shown together to highlight the opposing effects of movie content on amygdala BOLD signal levels. At the CAPS score of 62.5, predicted signal for the two movies is identical. The 95% CI were calculated separately for the two movies, and this accounts for the extensive overlap. A more accurate impression of the difference in mean amygdala BOLD is provided by the difference depicted in Fig 1D. **D)** Mean amygdala BOLD signal during more-arousing movie minus that during the less-arousing movie (dots), predicted mean amygdala signal (line), and predicted 95% CI (dark gray) plotted against CAPS scores (*r* = -0.46, *p* = 0.00089).

Consistent with what the standard model predicts, mean amygdala signal during the less-arousing movie increased with CAPS score [*r* = 0.40, *p* = 0.0041 **(Fig 2A and 2C and S3 Movie)**].

Surprisingly, however, mean amygdala signal during the more-arousing movie *decreased* with CAPS score [*r* = -0.29, *p* = 0.039 **(Fig 2B and 2C and S4 Movie)**].

We investigated this surprising result further by dividing subjects into quartiles by CAPS score. In subjects with low levels of symptoms (n = 13; bottom quartile, CAPS ≤ 26), mean amygdala signal during the more-arousing movie was significantly higher than during the less-arousing movie [*t*(12) = 2.22, *p* = 0.047], resulting in a positive difference score. In the highly symptomatic subjects (*n* = 13; top quartile, CAPS ≥ 78), the results were reversed so that signal during the more-arousing movie was significantly *lower* than signal during the less-arousing movie [*t*(12) = -2.51, *p* = 0.027].

Similar results were found when subjects were divided categorically into those with and those without PTSD **(S6 Text)**.

### Correlation between amygdala and ACC

#### ROI-Level

**Fig 3:**
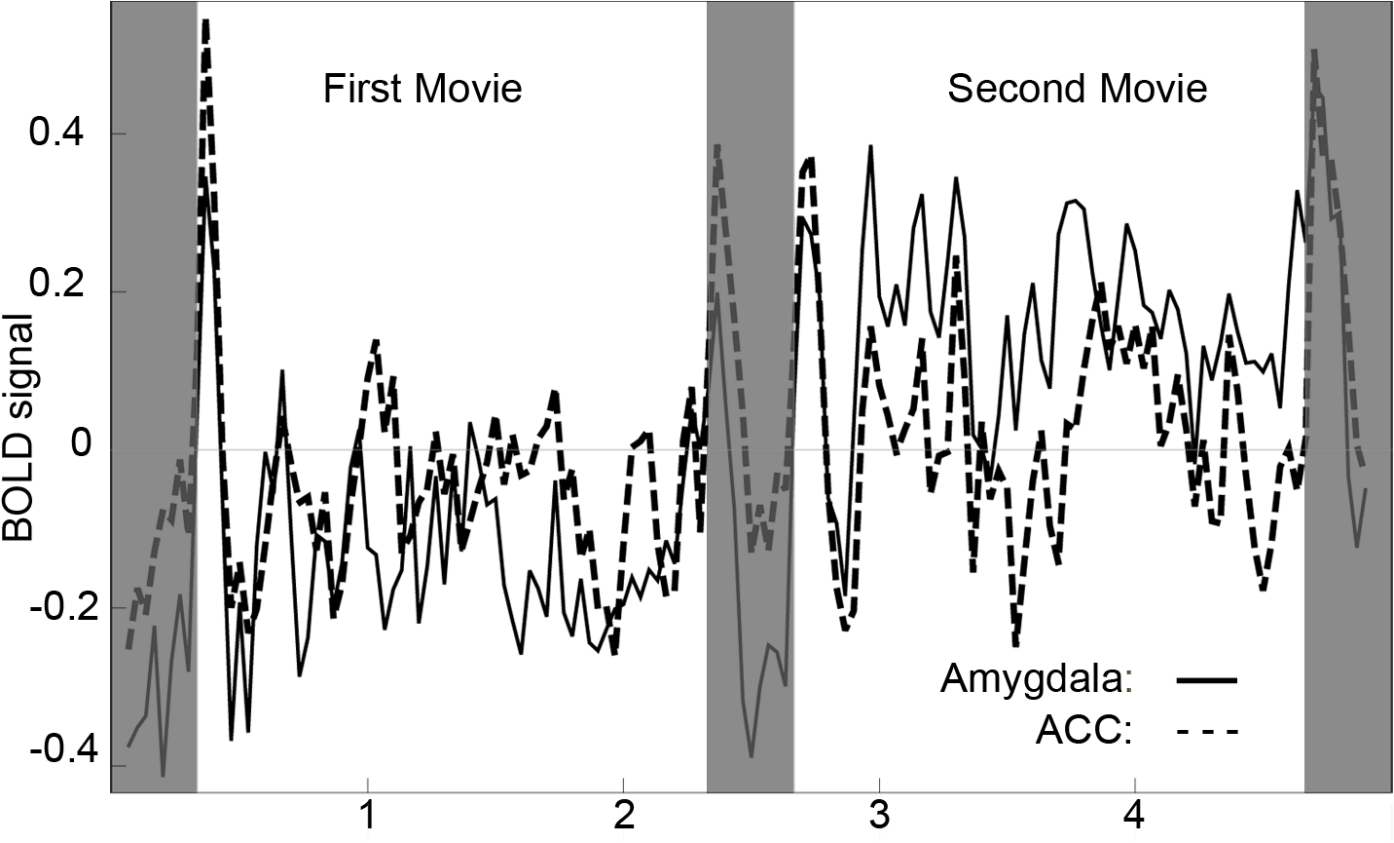
Correlation of amygdala and ACC BOLD time-courses. Mean ROI BOLD signal time courses of the amygdala and ACC averaged over all 50 subjects, regardless of the order in which they viewed the two movies. BOLD signal shown in arbitrary units; the 3 fixation periods indicated by shading. The Pearson correlation coefficient between these two averaged time courses is 0.62.

We calculated the Pearson correlation coefficient (hereafter referred to simply as “correlation”) between the BOLD signal time course averaged over the bilateral amygdala ROI and the BOLD signal time course averaged over the bilateral ACC ROI in each subject. The similarity of the time courses from the two ROIs is immediately apparent on visual inspection (Fig 3). Correlations between the two ROIs were positive in all subjects (mean *r* = 0.49, min 0.09, max 0.75). Because the correlation between the two ROIs was positive in every subject, the group-level statistic was highly significant (sign test, *p* < 5 x 10^−15^).

#### Voxel-level

**Fig 4:**
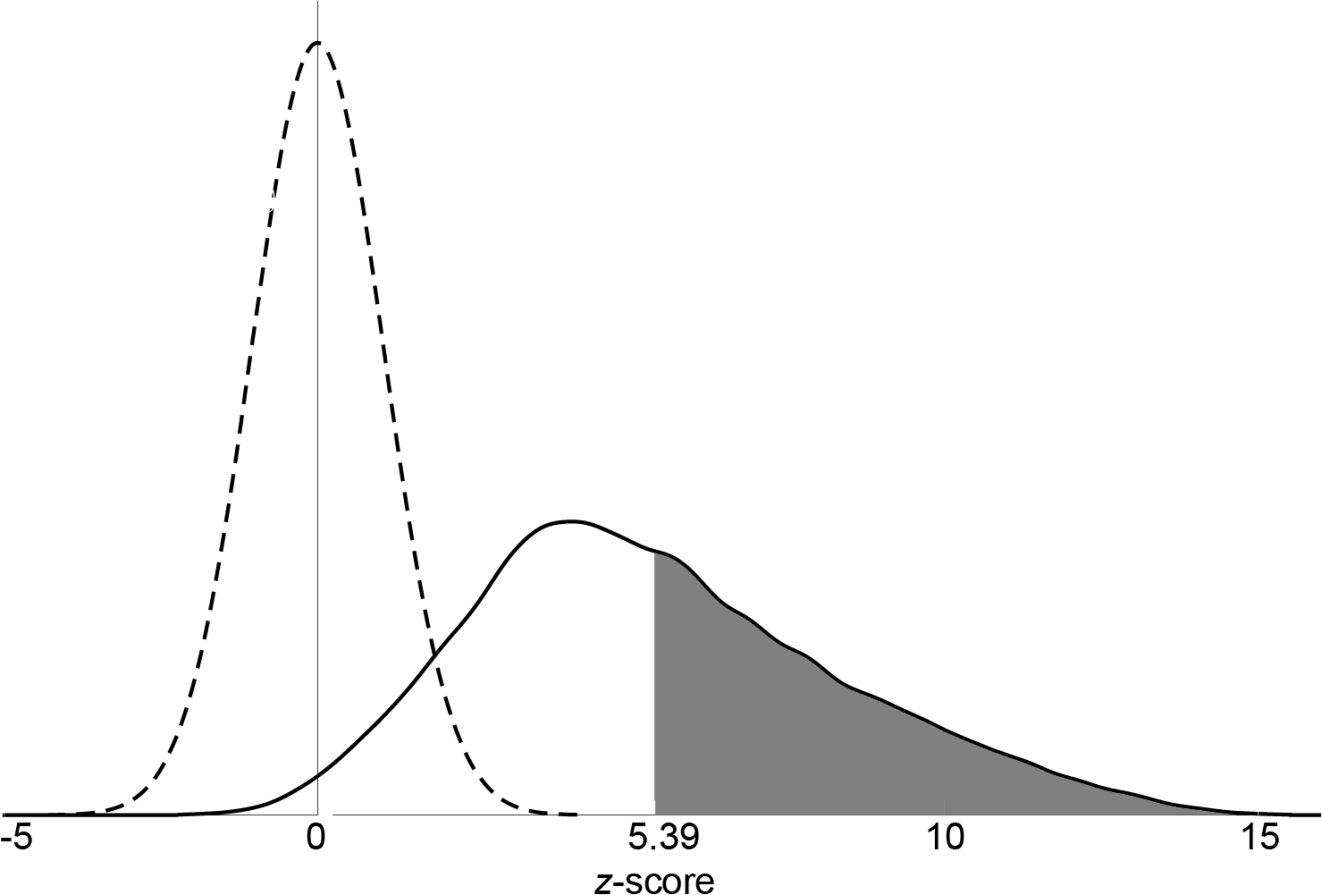
Distribution of all voxel-level BOLD time-course correlations between amygdala and ACC (as *z*-scores). Shown is the cumulative distribution function (CDF) for all amy_m_::acc_n_ voxel-level time-course correlations (solid black line) after being transformed into *z*-scores. Shaded region denotes *z*-scores > 5.39, considered significant by Bonferroni correction. Dashed line indicates CDF of null distribution *z*-scores (see Methods).

Next, we looked at correlations between the two ROIs at a finer spatial resolution: that of the individual voxels. For this, we calculated correlations in each subject between every possible amy_m_::acc_n_ voxel pair (see Methods). We then used these values to calculate a group-level *z*-score for each voxel pair, testing whether that pair’s correlation coefficient across subjects was significantly different from zero. This resulted in 724,880 *z*-values. Of these, 300,028 (~41%) were significantly greater than zero at the stringent two-tailed *z*-score of ±5.39, Bonferroni corrected for the large number of comparisons (Fig 4). These significant positive voxel-pair correlations involved more than 83% of voxels in each ROI (right amygdala: 99.6%, left amygdala: 94.6%, right ACC: 83.7%, left ACC: 82.7%), including 24.5% of the subgenual voxels on the left and 7.0% of the subgenual voxels on the right.

**Fig 5:**
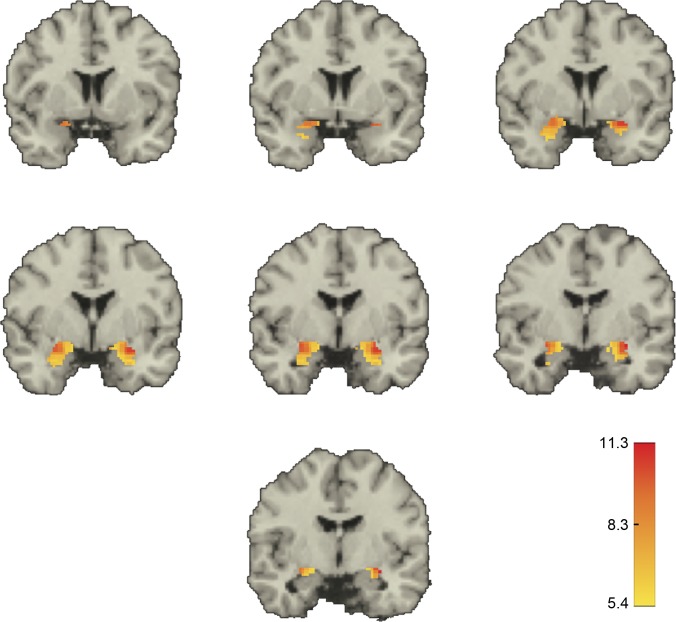
Voxel-level correlations in the amygdala. Shown in color are the median *z*-scores for each amygdala voxel whose BOLD-signal time course was significantly correlated with that of at least one voxel in the ACC in the same hemisphere. Threshold for significance was a *z*-score > 5.39. Medians were taken for each voxel over all the significant *z*-scores for that voxel. Only voxels within the amygdala are depicted. Coordinates are in MNI space with (0, 0, 0) at the anterior commissure. Coronal slices through the brain at y = 4, 2, 0, -2, -4, -6, and -8 mm showing the amygdala bilaterally. Positive y values are anterior to the anterior commissure.

**Fig 6:**
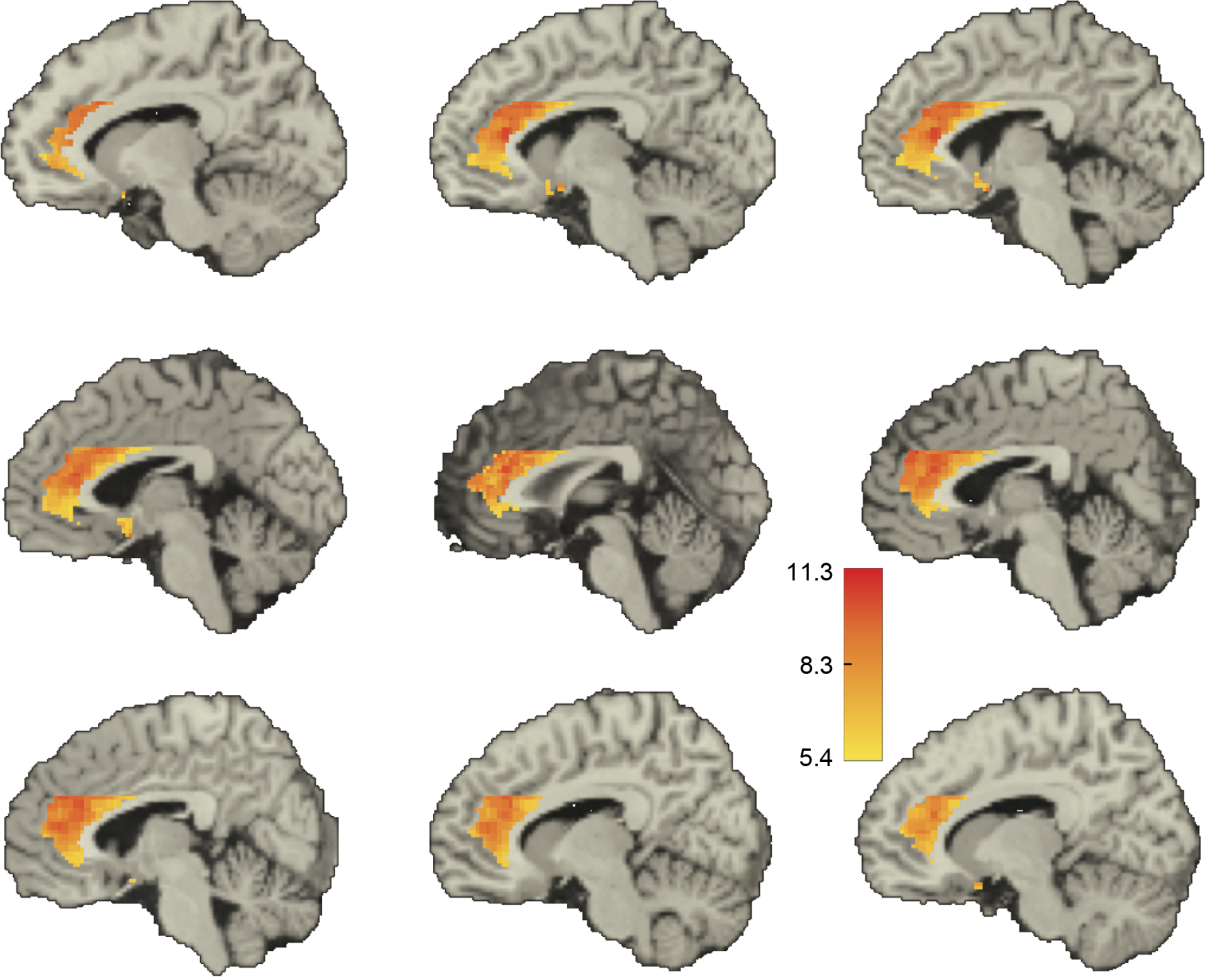
Voxel-level correlations in the ACC. Shown in color are the median *z*-scores for each ACC voxel whose BOLD-signal time course was significantly correlated with that of at least one voxel in the amygdala in the same hemisphere. Threshold for significance was a *z*-score > 5.39. Medians were taken for each voxel over all the significant *z*-scores for that voxel. Only voxels within the ACC are depicted. From upper left of figure: sagittal slices at x = 10, 6, 4, 2, -2, -4, -6, -10, and -12 mm. Positive x values are in the left hemisphere.

Qualitatively, we found a dorsal-ventral decreasing gradient in the median value of the significant positive correlations, both in the amygdala and in the ACC (Figs 5 and 6). Further, in the amygdala there also appeared to be a lateral-medial decreasing gradient. A similar pattern was obtained when we displayed for each voxel in one ROI the fraction of voxels in the other ROI with which it was significantly correlated **(S6 and S7 Figs)**.

In contrast to the widespread distribution of significant positive correlations, there was not a single significant negative correlation between any voxel in the ACC and any voxel in the amygdala. The closest the negative correlations came to the criterion *z*-score of -5.39 were a value of *z* = -3.29 (20 voxel pairs) on the right and *z* = -3.73 (3 voxel pairs) on the left.

### Relation of amygdala::ACC correlations to CAPS scores

#### ROI-Level

Although the correlation between the amygdala and ACC was positive in each subject, the correlations were less positive as CAPS scores increased (*r* = -0.34, *p* = 0.015).

#### Voxel-level

**Fig 7:**
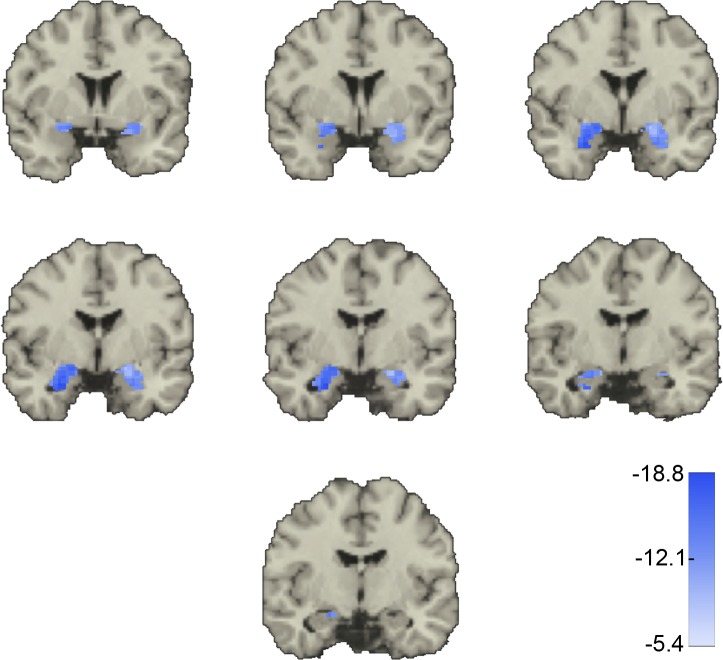
Amygdala voxel correlations with ACC whose values *decrease* as symptoms of post-combat emotional distress increase. For each correlation between one amygdala and one ACC voxel in the same hemisphere (amy_m_::acc_n)_, we regressed its value across all subjects with CAPS score. The majority of these regression values were negative. That is, as CAPS scores increased, the correlation between voxels in the amygdala and ACC decreased. Shown are the median of significant *z*-scores for voxels in the amygdala only. Threshold for significance was a *z*-score < -5.39. Coronal slices through the brain at y = 4, 2, 0, -2, -4, -6, and -8 mm showing the amygdala bilaterally. Only values within the amygdala ROI are shown.

**Fig 8:**
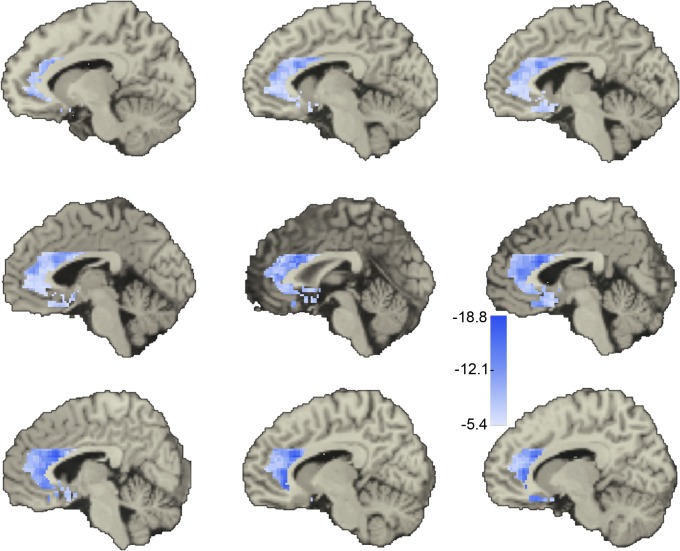
ACC voxel correlations with amygdala whose values *decrease* as symptoms of post-combat emotional distress increase. For each correlation between one ACC and one amygdala voxel in the same hemisphere (amy_m_::acc_n)_, we regressed its value across all subjects with CAPS score. The majority of these regression values were negative. That is, as CAPS scores increased, the correlation between voxels in the amygdala and ACC decreased. Shown are the median of significant *z*-scores for voxels in the ACC only. Threshold for significance was a *z*-score < -5.39. Only values within the ACC ROI are shown. From upper left of figure: sagital slices at x = 10, 6, 4, 2, -2, -4, -6, -10, and -12 mm.

**Fig 9:**
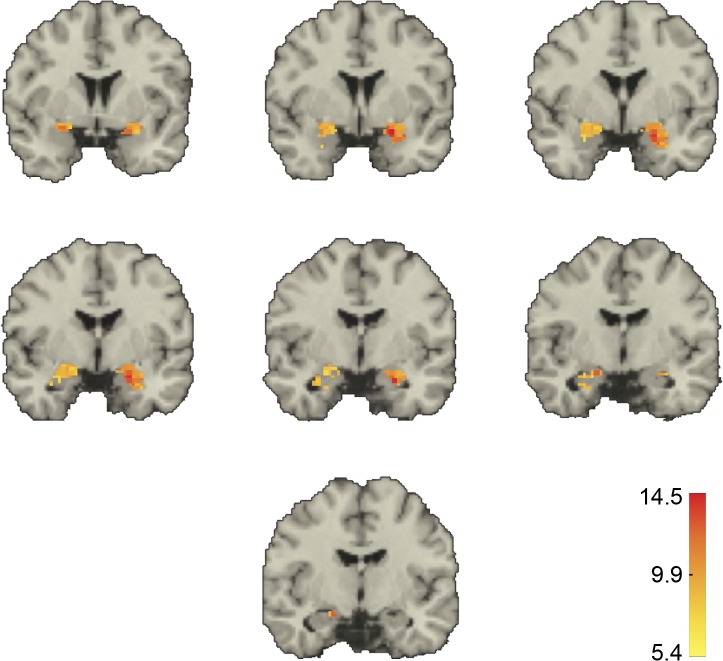
Amygdala voxel correlations with ACC whose values *increase* as symptoms of post-combat emotional distress increase. For each correlation between one amygdala and one ACC voxel in the same hemisphere (amy_m_::acc_n)_, we regressed its value across all subjects with CAPS score. Here we show the median of the positive regression values in the amygdala. Note that we calculated the correlations of all possible permutations between amygdala and ACC voxels, and so each voxel has many correlation values associated with it. Because of this, a voxel can have some correlations that are positively related to CAPS score and other correlations that are negatively related to CAPS score. Comparison of Fig 7 to Fig 9 and of Fig 8 to Fig 10 suggest that the distribution of those correlations that are negatively related to CAPS are different from those that are positively related to CAPS. Threshold for significance was a *z*-score > 5.39. Coronal slices through the brain at y = 4, 2, 0, -2, -4, -6, and -8 mm showing the amygdala bilaterally. Only values within the amygdala ROI are shown.

**Fig 10:**
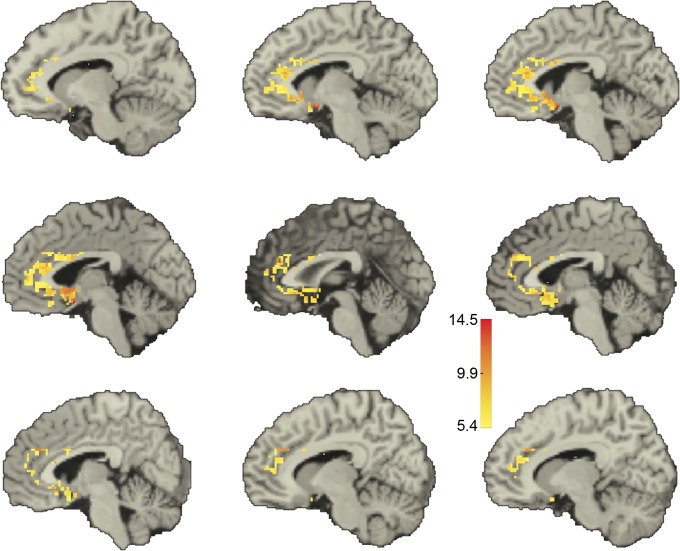
ACC voxel correlations with amygdala whose values *increase* as symptoms of post-combat emotional distress increase. For each correlation between one ACC and one amygdala voxel in the same hemisphere (amy_m_::acc_n)_, we regressed its value across all subjects with CAPS score. Here we show the median of the positive regression values in the ACC. Threshold for significance was a *z*-score > 5.39. From upper left of figure: sagital slices at x = 10, 6, 4, 2, -2, -4, -6, -10, and -12 mm. Only values within the ACC ROI are shown.

Approximately 54% of the amy_m_::acc_n_ voxel pair correlations decreased significantly (were less positive) with increasing CAPS score. These pairs included voxels throughout the ACC and amygdala and were more numerous on the right than the left (Figs 7 and 8). Approximately 5.3% of the amy_m_::acc_n_ voxel pair correlations increased significantly (were more positive) with CAPS scores (Figs 9 and 10). Although these pairs included voxels throughout the ACC and amygdala, this 5% was most heavily concentrated in the sub-genual region of the left ACC and the dorsal portion of the left amygdala.

## Discussion

SCR magnitudes were significantly larger during the combat movie than during the civilian movie, and this effect was not correlated with CAPS scores. Because SCRs are often taken as indices of arousal, this suggests that subjects found the combat movie to be more emotionally arousing regardless of the severity of their post-combat emotional distress. This is not surprising, as a meta-analysis of physiologic measures in PTSD concluded that altered SCR reactivity was not a robust index of PTSD symptomatology [69]. This finding also suggests that CAPS-related differences in amygdala BOLD signal are not the result of differential levels of stimulus-induced arousal. It is worth repeating here that the combat or civilian setting is only one of a number of characteristics that differ between the two movies (see Limitations below). The finding that some subjects showed increased SCRs with decreased BOLD signal in the amygdala is consistent with reports from fMRI [70] and brain-lesion [71,72] studies demonstrating that the SCRs can exist in the absence of increased amygdala activity.

Our fMRI results can be organized under three main findings. First, we found evidence that post-combat emotional distress can be associated not only with increases but also with decreases in amygdala reactivity. Second, we found wide-spread, exclusively positive correlations between BOLD signal in the ACC and that in the amygdala. Third, using CAPS scores as a measure, participants with higher levels of emotional distress had lower correlations (less positive, but still not negative) between BOLD signal in the amygdala and that throughout much of the ACC.

We will now elaborate on these three main findings in the context of current thought about the underlying neural changes that go along with PTSD. We will also touch on current thought on the relationship between the ACC and the amygdala in non-clinical populations.

### First Finding: Bi-directional Changes in Amygdala Reactivity

We found evidence that amygdala responses can be altered bi-directionally in the same person in a stimulus-dependent manner. For the less-arousing movie, amygdala reactivity increased with CAPS scores. For the more-arousing movie, amygdala reactivity *decreased* with CAPS scores. Compared to subjects with lower CAPS scores, subjects with higher CAPS scores had both a greater *and* a lesser amygdala response depending on the stimulus.

The standard neurocircuitry model of PTSD has been structured around the early finding that subjects with PTSD can display increased amygdala reactivity to trauma-related stimuli [50,73]. This finding was confirmed in many subsequent reports [30,35] that have also extended it to include stimuli that are not explicitly trauma-related [31,32]. The standard model posits that amygdala hyper-reactivity contributes heavily to the symptoms of PTSD. The fact that not all studies have found evidence of PTSD-related amygdala hyper-reactivity has been attributed to methodological issues (e.g. PET vs. fMRI) [46], amygdala habituation [74], sample size [45], inadequate symptom provocation [47], and heterogeneity in subject populations [75]. The accuracy of the basic tenet that increased amygdala reactivity underlies the symptoms of PTSD has not itself been questioned. In this report, we presented evidence of stimulus-dependent *decreases* as well as increases in amygdala reactivity in the same subjects.

CAPS scores can be broken down into three sub-scales: intrusion, avoidance/numbing, and arousal. Decreased amygdala reactivity during the more-arousing movie did not appear to depend on one particular cluster of symptoms. For example, it might have been thought that subjects with high levels of avoidance or low levels of intrusion would be more likely to have decreased amygdala activation. There was no evidence, however, for a differential loading of any of the three CAPS subscales with decreased amygdala BOLD signal during the more-arousing movie. Neither did the decreased amygdala reactivity appear to reflect decreased emotional arousal as measured by SCRs. The relation between decreased amygdala reactivity and subjective experience has yet to be elucidated.

### Second Finding: Amygdala correlation with ACC

Along with the hypothesis that increased amygdala reactivity leads to symptoms of PTSD, the standard model proposes both a mechanistic explanation for the increase. In the mechanistic component of the standard model, amygdala hyperactivity reflects a deficiency in top-down inhibitory regulation from the ACC. This line of thought draws from neuroimaging evidence of an inverse relationship between activity in the amygdala and that in areas of the medial frontal cortex mPFC [3,4,31,32,75], the ACC being the area cited most consistently. The thinking here is that if healthy control subjects show increased ACC activity along with decreased amygdala activity, while subjects with PTSD show decreased ACC activity simultaneous with increased amygdala activity, perhaps the ACC exerts an inhibitory effect on the amygdala. If this were the case, then increased activity in the ACC would cause decreased activity in the amygdala. In short, subjects with PTSD are thought of as having a relative lack of ACC activity and, thus, a relative lack of inhibitory dampening of the amygdala’s reactivity.

There are two ways in which this mechanistic explanation may be critiqued: A) One can challenge the ability of the data on which it is based to support the inferences drawn from them. B) One can present findings that either fail to replicate or, more strongly, findings that contradict the data.

A) In terms of the ability of prior research’s data to support the inferences underlying the standard model’s mechanistic explanation, it is well known that fMRI and related neuroimaging techniques such as positron emission tomography (PET) are not currently able to make strong statements about inter-regional regulation of neural activity. Signal in these techniques provides only an indirect representation of neuronal electrical activity [76,77]. Also, these signals are not able to distinguish between the electrical activity of excitatory neurons and that of inhibitory neurons. This critique is not new, and every technique has its strengths and weaknesses. As long as these are kept in mind, it is reasonable to use neuroimaging data to formulate hypotheses on the workings of neural circuitry.

B) Our results are not consistent with data on which the standard model is based. Not only did we fail to find a negative correlation between signal anywhere in the amygdala and signal in even a small portion of the ACC, we also found widespread positive correlations in signal between these two regions. Although this is not the first report of a positive correlation between the amygdala and ACC in subjects with trauma exposure [49–51], it is the most extensive. We examined BOLD signal correlations between every possible amygdala::ACC voxel pair in a task that elicited clear stimulus-dependent and symptom-dependent changes in amygdala activation. We do not interpret our results as evidence of top-down excitation. It is quite possible, for example, that both regions are responding to common input that synchronizes their activity. We do point out, though, that our data stand in direct contradiction to prior data that has frequently been used to support the mechanistic model of a top-down inhibition.

### Third Finding: Effect of symptom severity on the correlation between amygdala and ACC

Many prior investigations have held the implicit assumption that the degree of correlation between the amygdala and the ACC is not changed in those with PTSD. That is, changes in the amygdala’s reactivity were thought to depend on changes in ACC activity rather than on changes in the degree to which activity in the two regions is correlated. There is recent evidence, though, that the strength of the correlation between the two areas can change in proportion to perceived threat after combat experience [78]. Our results indicate that the strength of the correlation between the amygdala and ACC can vary with symptom severity as well. As CAPS scores increased, correlation between amygdala and ACC BOLD signal decreased in more than half of the voxel-pairs and increased in about 5% of the voxel pairs.

It is not yet known whether either the ACC or amygdala exert a direct influence on the other’s activity. Nor is it known whether an improvement in a subject’s CAPS scores would be caused by or associated with an increased correlation between the amygdala and the ACC. Would it be possible, for example, to improve post-combat symptoms by strengthening correlated activity between the amygdala and ACC? This latter issue may offer a treatment approach and is a matter for future research.

### Dorsal versus ventral ACC in clinical and non-clinical populations

A number of researchers interested in clinical as well as in non-clinical populations have proposed a functional distinction between the dorsal and the ventral ACC. This distinction has been characterized in several ways. Of relevance to our results, it has been proposed that activity in the dorsal ACC may excite the amygdala, while activity in the ventral ACC may inhibit activity in the amygdala [79]. If such a mechanism exists and if excitation and inhibition are reflected in positive and negative correlations in BOLD signal, respectively, our results may be seen by some as being consistent with this distinction. While we did not find any significant negative correlations, we did find a decrease in the number and magnitude of the positive correlations from the dorsal to the ventral regions of the ACC. Our study was not designed to separate dorsal from ventral ACC, and we present our results in a descriptive rather than statistical manner.

### Limitations of our study

One unavoidable limitation of this study stems from the fact that the results presented here appear to challenge the prevailing conceptualization of how trauma affects neural functioning. It would be injudicious to rely on the results from just one study to completely abandon an accepted model or to propose a new one. We do believe, though, that these results remind us that all models of human brain function should be viewed as tentative, heuristic simplifications of an extremely complex system.

A further limitation of our study is the lack of subjective ratings of participants’ emotional experiences while watching the movies. We can not, therefore, directly relate BOLD signal changes with specific emotional states. However, we recorded SCRs, and SCRs have been used for well over 50 years as a measure of emotional activation and arousal [64]. Another limitation is that although the use of movies as stimuli provided several benefits, including what has been termed “ecological validity” [80], such complex stimuli also present challenges and drawbacks. Because of the marked variation over time of the images and sound, there is no accepted method for matching the low-level perceptual features of one movie to those of another. In fact, one of the reasons that experimental stimuli are typically less naturalistic is that less-naturalistic stimuli are easier to control and model. Our results do not isolate the features of the combat movie that led to the difference between participants’ reactions to it versus the civilian movie. Nor can we isolate what features of either movie caused participants with greater CAPS scores to respond differently than those with lower CAPS scores. Finally, we do not have similar measures of BOLD signal in these subjects before they were deployed. We cannot, therefore, determine the degree to which the findings are indicative of dispositional tendencies or a result of combat stress.

In conclusion, our results suggest that symptoms of post-traumatic emotional distress can be associated with decreased as well as increased amygdala reactivity. These bi-directional changes can occur in close succession in the same person. We found evidence that bi-directional changes in amygdala reactivity can be positively correlated with activity throughout the ACC. The proposal that PTSD results from decreased top-down inhibition of the amygdala by the ACC has been based in large part on prior findings of a negative correlation between these two regions. Finally, we found that a linear function can describe symptom-related changes in amygdala responses throughout a broad range of symptom severity. It remains to be seen whether similar results will be found in other experimental paradigms or with emotional disturbances following non-combat stresses.

## Supporting Information

S1 Fig Schematic depiction of two possible results for the correlation of amygdala BOLD signal and CAPS scores during the two movies. In both possibilities, we predicted that amygdala BOLD signal would be greater during the civilian movie than during the combat movie for all subjects. We also predicted in both possibilities that amygdala BOLD signal would increase with increasing CAPS scores during both movies. What distinguishes the result depicted in the two panels is that in panel A the function relating CAPS score and amygdala BOLD signal is the same for both movies. In panel B, amygdala BOLD signal increases more rapidly with increasing CAPS scores during the combat movie than during the civilian movie. Clearly there are other possible results. For example, amygdala BOLD signal could decrease with CAPS scores during either or both movies. We did not hypothesize that this would be the case.
(TIF)

S2 Fig Graphic representation of the measurement of SCR areas. The x-axis displays time in seconds. The y-axis displays the value of the skin conductance in micro-siemens (μS). The skin conductance tracing shown here is from one of the subjects in the study. It was chosen because it depicts several aspects of the measurement process. It shows the areas of six SCRs depicted as gray rectangles. Blue dots indicate the start of the SCR. Red dots indicate the maximum value of the SCR, which is by definition located at the peak. SCR height is defined as the difference between the value at the peak and the value at the start. Black dots indicate the half-maximum values before and after the peak. SCR width is defined as the interval between the two half-maximum values. SCR area is defined as the product of the height and the width. Note the following features. 1) We required SCR heights to be greater than 0.05 μS. The skin conductance tracing shows two instances of what appear to be SCRs neither of which has a height greater than 0.05 μS. One of these follows the second SCR, and the other is located immediately after the peak of the sixth SCR. 2) An SCR sometimes occurs superimposed upon another SCR. In this tracing, the fifth SCR starts before the fourth SCR has returned to its initial value. When this happens, the descending curve for the first of the two SCRs is extrapolated from its peak. The half-maximum point following the peak is then estimated to occur along the extrapolated portion of this curve. The half-maximum point of the sixth SCR shown here is also located on an extrapolated portion of the curve. This occurred because of the superimposition of a small upward deflection less than 0.05 μS immediately after the peak value.
(TIF)

S3 Fig Define and model a null-distribution. We used randomized sign-permutations of voxel time courses to establish a reasonable null distribution. Here, the null distribution tests the null hypothesis (H_0_) that signal time courses in one ROI (but not both) can be sign-permuted in a subset of subjects without affecting the statistics of the distribution of amy::ACC correlations. The advantage of the sign-permutation method is that it retains signal temporal autocorrelation and spatial distribution across the ROIs, while systematically removing any correlation (should it exist) from varying subsets of subjects. If the null hypothesis is correct, there is no correlation between the amygdala and the ACC; sign permutation would therefore not alter the statistics. The degree to which sign permutation alters the results then provides a reasonable measure of the correlation between the two ROIs. To accomplish this, we performed 10,000 iterations in which the entire amy_M_::acc_N_ correlation matrix was multiplied by either 1 or -1 with an equal probability. This was done separately for each hemisphere. This is exactly equivalent to inverting the sign of the signal time course in each of the voxels of either the amygdala or ACC (but not both) and then recalculating the correlations. It is, however, far less computationally intensive. A graphic depiction of this process is shown in this figure.
(TIF)

S4 Fig Model the statistics of the null-distribution. Statistics of the Null Distribution: Next, for each iteration, we calculated single-sample *t*-scores for each amy_m_::acc_n_ voxel pair, yielding a final total of 10,000 *t*-matrices for the null distribution. Each resulting *t*-matrix provided an estimate of whether the mean correlation we could expect in each amy_m_::acc_n_ pair averaged over 50 subjects would be significantly different from zero if the sign of the signal time course did not matter. This process is depicted in the right-hand column of S3 Fig and in this figure.
(TIF)

S5 Fig Compare statistics of real data to null-distribution data. Compare statistics of real data and null-distribution data: We were then able assign each amy_m_::acc_n_ *t*-score in our actual data a *z*-score based on the 10,000 null-distribution *t*-matrices. We did not pool the *z*-statistic across voxels because there appeared to be a periodic spatial variation in the values of the null-distribution *z*-scores. We set the strict criterion *z*-score for each voxel at ±5.39 (alpha = 0.05/724,880) to correct for the large number of comparisons. This process is shown in the bottom image of S5 Fig.
(TIF)

S6 Fig Depiction of the connectedness of the amygdala with the ACC. Color indicates the fraction of voxels in the ipsi-lateral ACC with which each voxel in the amygdala is significantly correlated. All significant correlations were positive. From the upper left, coronal slices through the brain at y = 4, 2, 0, -2, -4, -6, and -8 mm showing the amygdala bilaterally.
(TIF)

S7 Fig Depiction of the connectedness of the ACC with the amygdala. Color indicates the fraction of voxels in the ipsi-lateral amygdala with which each voxel in the ACC is significantly correlated. All significant correlations were positive. From upper left of figure: sagittal slices at x = 10, 6, 4, 2, -2, -4, -6, -10, and -12 mm.
(TIF)

S8 Fig Schematic representation of a subject viewing a movie. This schematic image indicates the general area of the amygdala in a cut-away view of the brain. The amygdala is colored to represent the correlation of BOLD activity with the ACC. This is done as in Figs 5 and 6.
(TIF)

S1 Text Use of bilateral amygdala for ROI. (DOC)

S2 Text Mean amygdala signal. (DOC)

S3 Text Similarity and differences of this method to boxcar convolution. (DOC)

S4 Text Regressors and “nuisance variables.” (DOC)

S5 Text Correlating CAPS scores with voxel-level inter-ROI BOLD-signal correlations. (DOC)

S6 Text Analysis with categorical division of subjects. (DOC)

S1 Movie (MP4)

S2 Movie (MP4)

S3 Movie Civilian movie clip with amygdala BOLD-signal time course. Clip taken from the beginning of the civilian movie. To provide a qualitative sense of the differences in amygdala response between participants with low CAPS scores and high CAPS scores, two separate amygdala BOLD-signal time courses are shown along with the movie. One is averaged over the 25 participants whose CAPS scores fall below the median (low-PTSD), and the other is averaged over the 25 participants whose CAPS cores fell above the median (high-PTSD). Visual comparison of these two suggests that, overall, both groups respond to the same stimuli, but that the participants with higher CAPS scores maintain a higher level of amygdala response throughout much of the movie than the participants with lower CAPS scores. To create these visual summaries, BOLD signal from each subject was standardized to have unit variance and zero mean before being averaged across subjects in each group. The average of the first five time points was then subtracted from the rest of the time course for each group. The y-axis, not shown because the units are arbitrary, is the same for both groups.
(MP4)

S4 Movie Combat movie clip with amygdala BOLD-signal time course. Clip from the beginning of the combat movie. To provide a qualitative sense of the differences in amygdala response between participants with low CAPS scores and high CAPS scores, two separate amygdala BOLD-signal time courses are shown along with the movie. One is averaged over the 25 participants whose CAPS scores fall below the median (low-PTSD), and the other is averaged over the 25 participants whose CAPS cores fell above the median (high-PTSD). Visual comparison of these two suggests that, overall, both groups respond to the same stimuli, but that the participants with higher CAPS scores maintain a lower level of amygdala response throughout much of the movie than the participants with lower CAPS scores. To create these visual summaries, BOLD signal from each subject was standardized to have unit variance and zero mean before being averaged across subjects in each group. The average of the first five time points was then subtracted from the rest of the time course for each group. The y-axis, not shown because the units are arbitrary, is the same for both groups.
(MP4)

S1 Table Comparison of Results For All Subjects to Male-Only Subjects. (DOC)

S2 Table Inclusion of additional predictive variables. (DOC)

S3 Table Analysis of first 27 and last 23 subjects separately. (DOC)

S4 Table Correlation coefficients of the three CAPS sub-scales and the SCR data. (DOC)
