# Supplementary figures and images for: Bi-Directional Tuning of Amygdala Sensitivity in Combat Veterans Investigated with fMRI
Source: PLoS One. 2015 Jun 29;10(6):e0130246. doi: 10.1371/journal.pone.0130246 (PMC4488265; doi:10.1371/journal.pone.0130246)

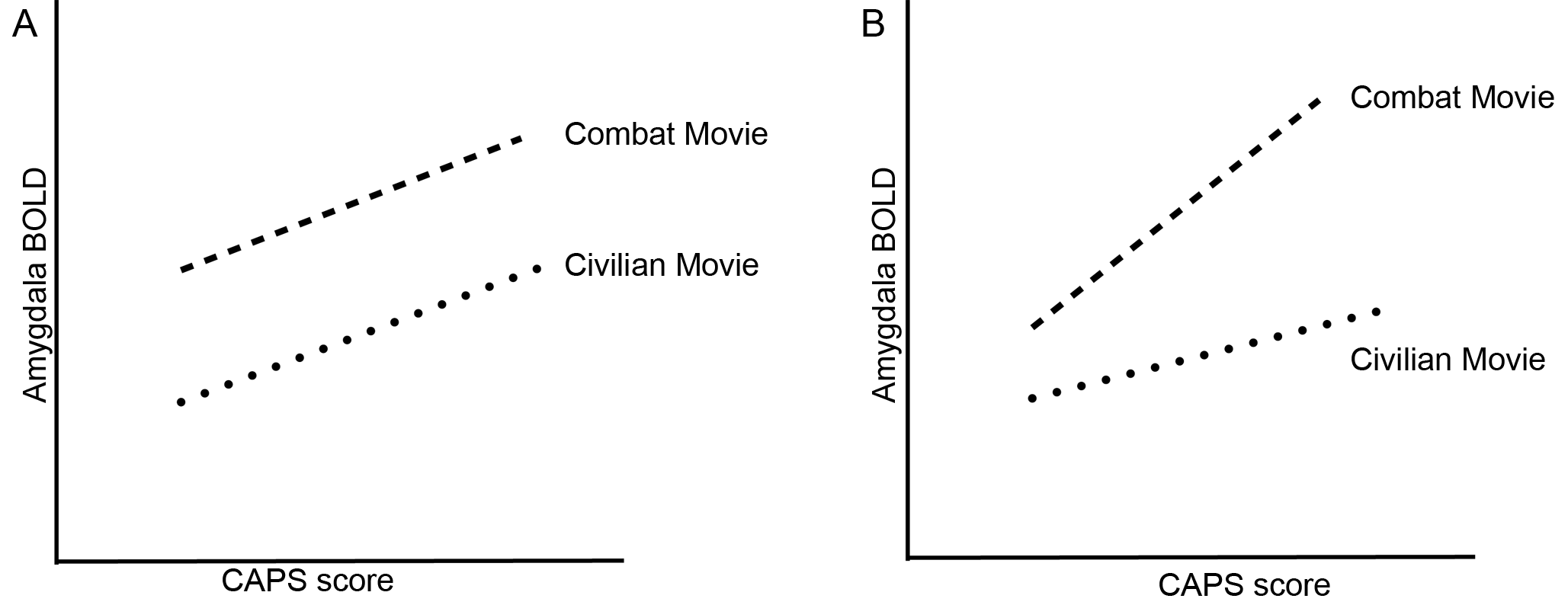

Supplement: S1 Fig — In both possibilities, we predicted that amygdala BOLD signal would be greater during the civilian movie than during the combat movie for all subjects. We also predicted in both possibilities that amygdala BOLD signal would increase with increasing CAPS scores during both movies. What distinguishes the result depicted in the two panels is that in panel A the function relating CAPS score and amygdala BOLD signal is the same for both movies. In panel B, amygdala BOLD signal increases more rapidly with increasing CAPS scores during the combat movie than during the civilian movie. Clearly there are other possible results. For example, amygdala BOLD signal could decrease with CAPS scores during either or both movies. We did not hypothesize that this would be the case. (TIF) [file pone.0130246.s001.tif]

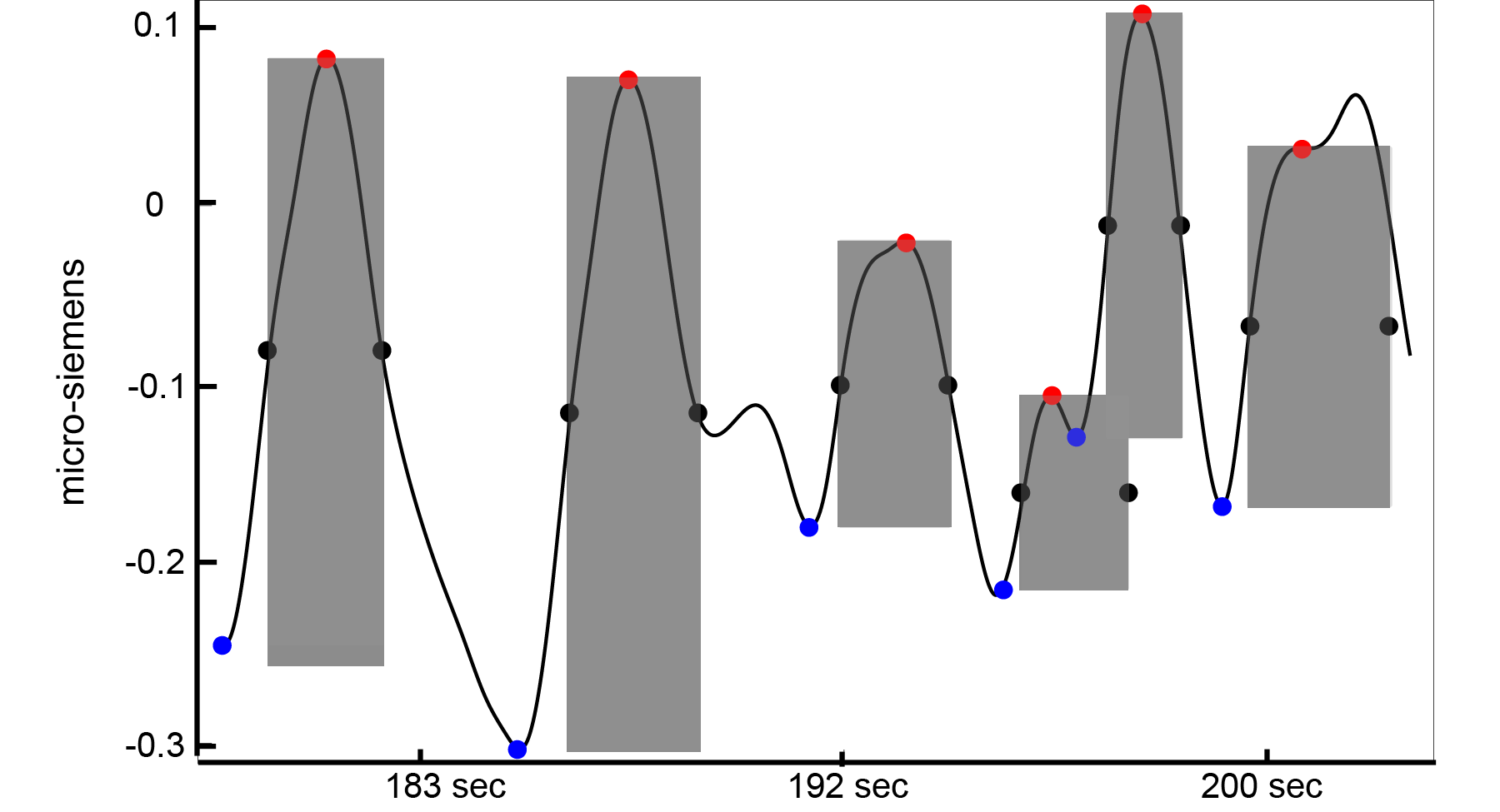

Supplement: S2 Fig — The x-axis displays time in seconds. The y-axis displays the value of the skin conductance in micro-siemens (μS). The skin conductance tracing shown here is from one of the subjects in the study. It was chosen because it depicts several aspects of the measurement process. It shows the areas of six SCRs depicted as gray rectangles. Blue dots indicate the start of the SCR. Red dots indicate the maximum value of the SCR, which is by definition located at the peak. SCR height is defined as the difference between the value at the peak and the value at the start. Black dots indicate the half-maximum values before and after the peak. SCR width is defined as the interval between the two half-maximum values. SCR area is defined as the product of the height and the width. Note the following features. 1) We required SCR heights to be greater than 0.05 μS. The skin conductance tracing shows two instances of what appear to be SCRs neither of which has a height greater than 0.05 μS. One of these follows the second SCR, and the other is located immediately after the peak of the sixth SCR. 2) An SCR sometimes occurs superimposed upon another SCR. In this tracing, the fifth SCR starts before the fourth SCR has returned to its initial value. When this happens, the descending curve for the first of the two SCRs is extrapolated from its peak. The half-maximum point following the peak is then estimated to occur along the extrapolated portion of this curve. The half-maximum point of the sixth SCR shown here is also located on an extrapolated portion of the curve. This occurred because of the superimposition of a small upward deflection less than 0.05 μS immediately after the peak value. (TIF) [file pone.0130246.s002.tif]

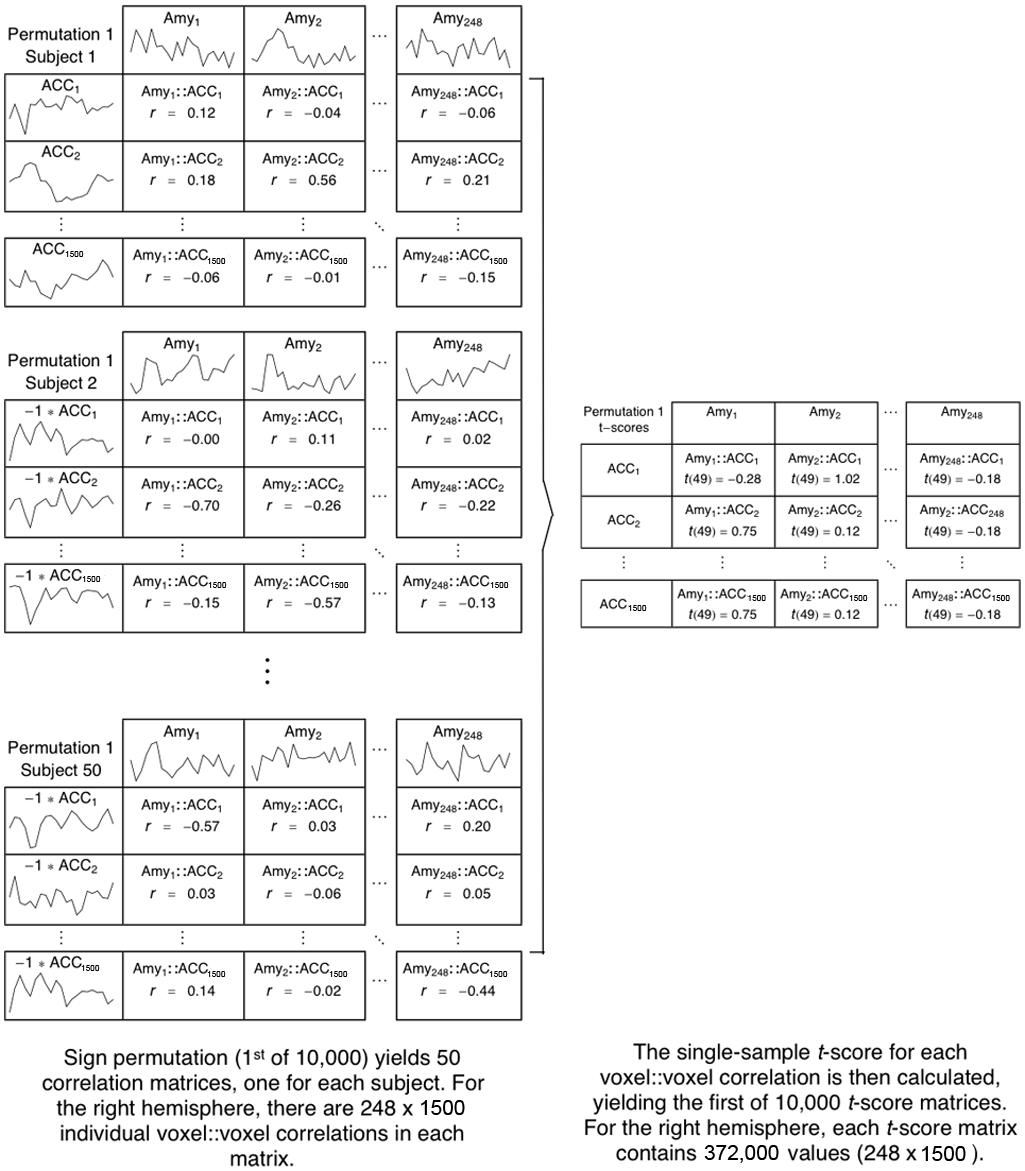

Supplement: S3 Fig — We used randomized sign-permutations of voxel time courses to establish a reasonable null distribution. Here, the null distribution tests the null hypothesis (H0) that signal time courses in one ROI (but not both) can be sign-permuted in a subset of subjects without affecting the statistics of the distribution of amy::ACC correlations. The advantage of the sign-permutation method is that it retains signal temporal autocorrelation and spatial distribution across the ROIs, while systematically removing any correlation (should it exist) from varying subsets of subjects. If the null hypothesis is correct, there is no correlation between the amygdala and the ACC; sign permutation would therefore not alter the statistics. The degree to which sign permutation alters the results then provides a reasonable measure of the correlation between the two ROIs. To accomplish this, we performed 10,000 iterations in which the entire amyM::accN correlation matrix was multiplied by either 1 or -1 with an equal probability. This was done separately for each hemisphere. This is exactly equivalent to inverting the sign of the signal time course in each of the voxels of either the amygdala or ACC (but not both) and then recalculating the correlations. It is, however, far less computationally intensive. A graphic depiction of this process is shown in this figure. (TIF) [file pone.0130246.s003.tif]

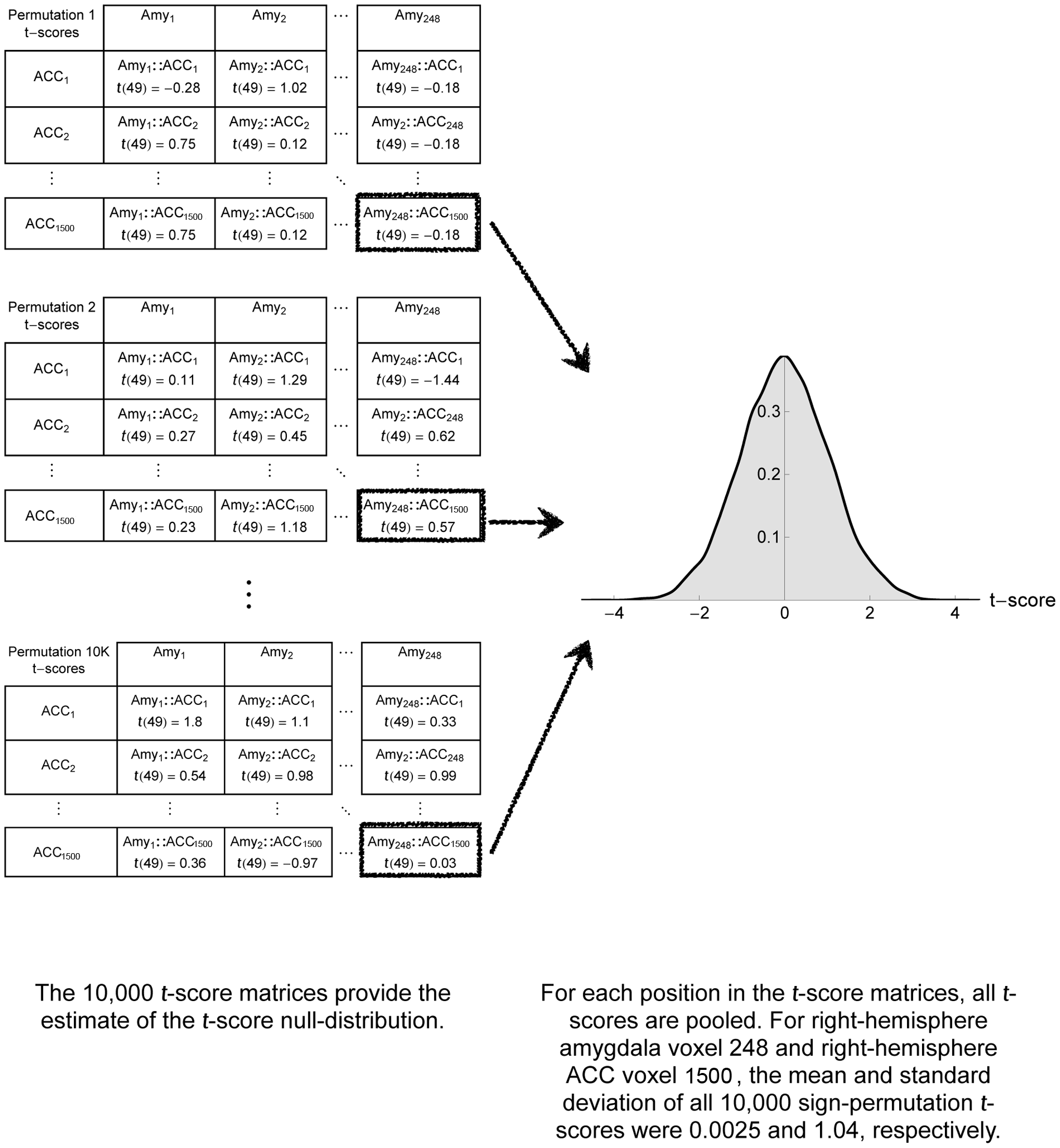

Supplement: S4 Fig — Statistics of the Null Distribution: Next, for each iteration, we calculated single-sample t-scores for each amym::accn voxel pair, yielding a final total of 10,000 t-matrices for the null distribution. Each resulting t-matrix provided an estimate of whether the mean correlation we could expect in each amym::accn pair averaged over 50 subjects would be significantly different from zero if the sign of the signal time course did not matter. This process is depicted in the right-hand column of S3 Fig and in this figure. (TIF) [file pone.0130246.s004.tif]

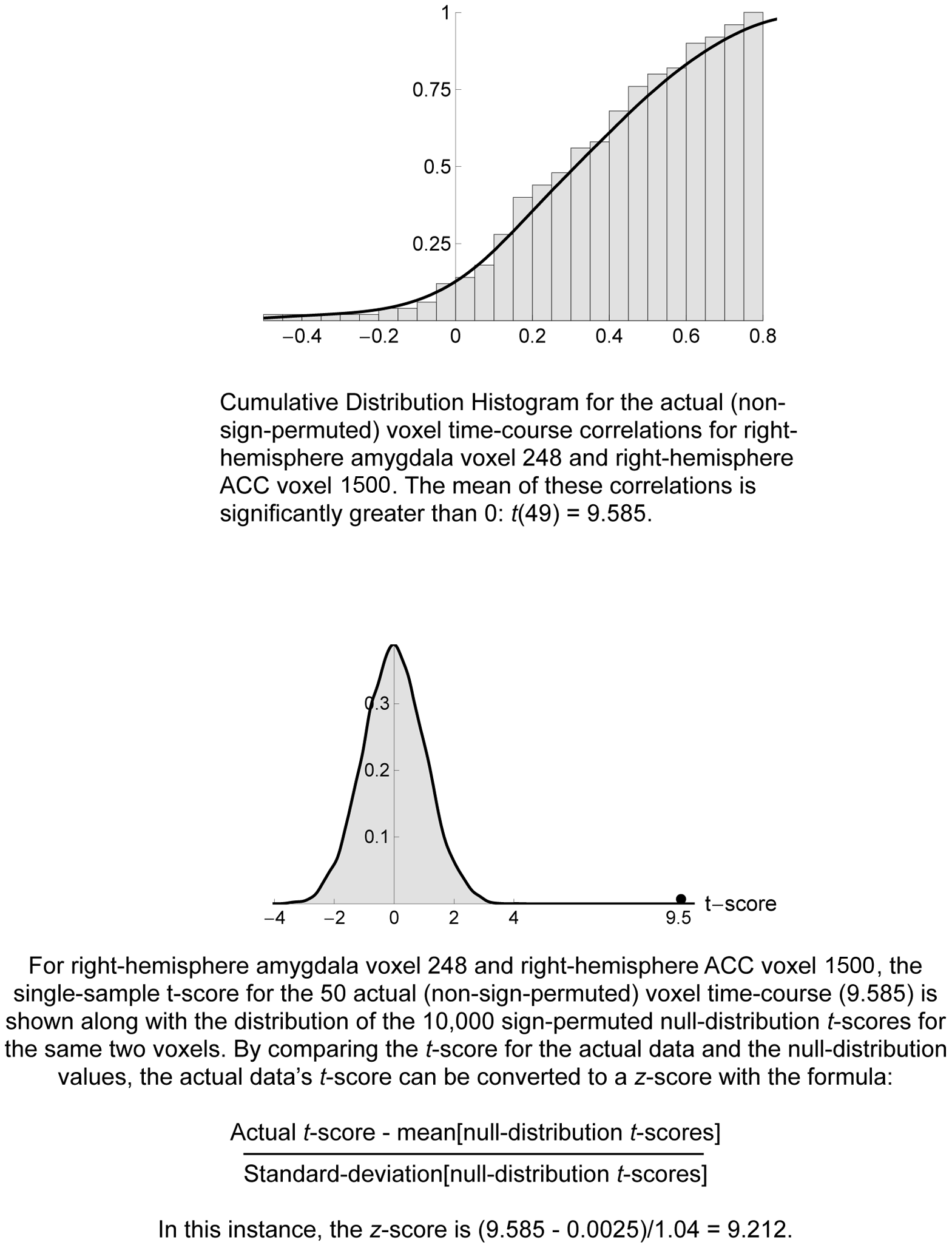

Supplement: S5 Fig — Compare statistics of real data and null-distribution data: We were then able assign each amym::accn t-score in our actual data a z-score based on the 10,000 null-distribution t-matrices. We did not pool the z-statistic across voxels because there appeared to be a periodic spatial variation in the values of the null-distribution z-scores. We set the strict criterion z-score for each voxel at ±5.39 (alpha = 0.05/724,880) to correct for the large number of comparisons. This process is shown in the bottom image of S5 Fig. (TIF) [file pone.0130246.s005.tif]

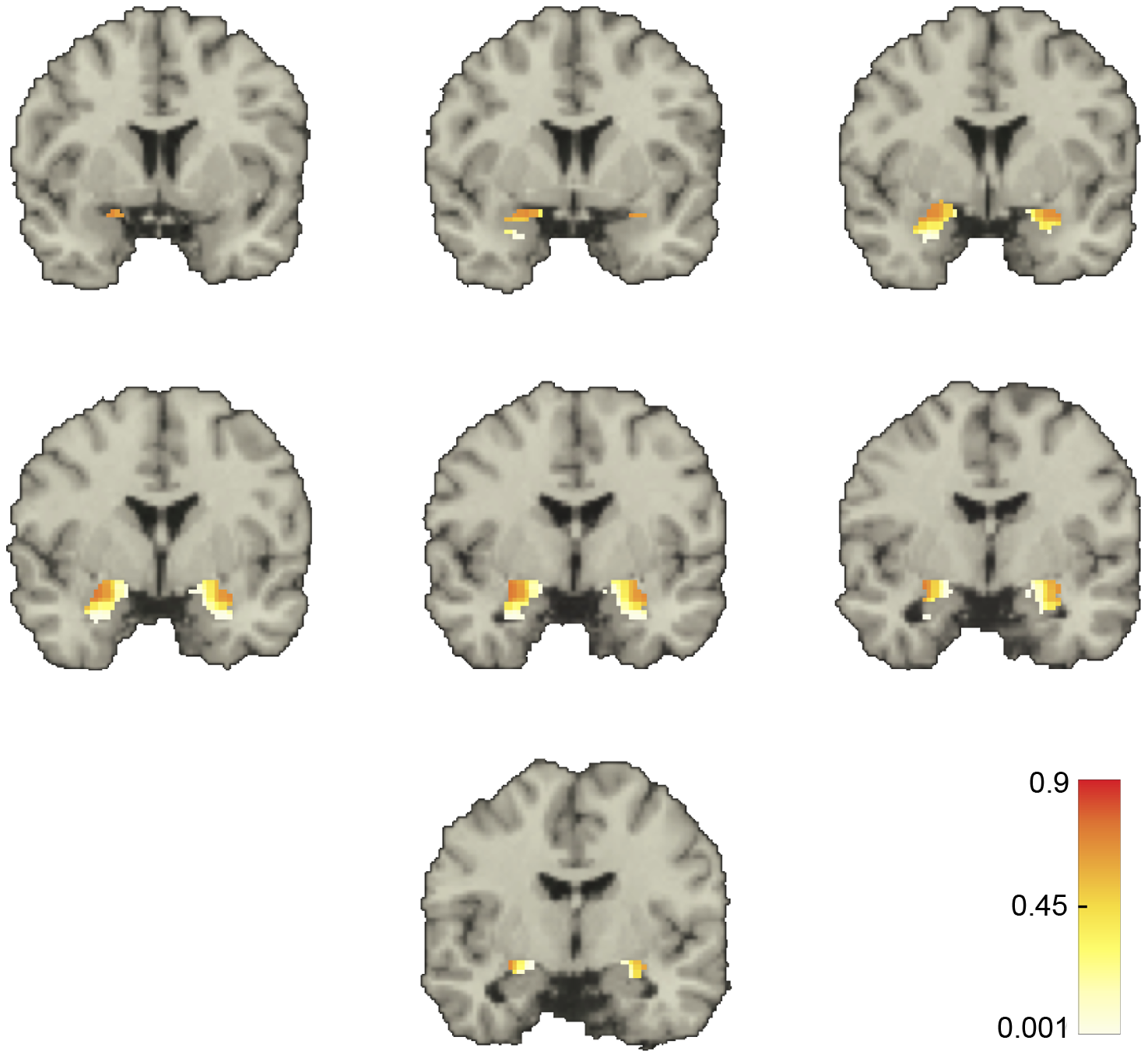

Supplement: S6 Fig — Color indicates the fraction of voxels in the ipsi-lateral ACC with which each voxel in the amygdala is significantly correlated. All significant correlations were positive. From the upper left, coronal slices through the brain at y = 4, 2, 0, -2, -4, -6, and -8 mm showing the amygdala bilaterally. (TIF) [file pone.0130246.s006.tif]

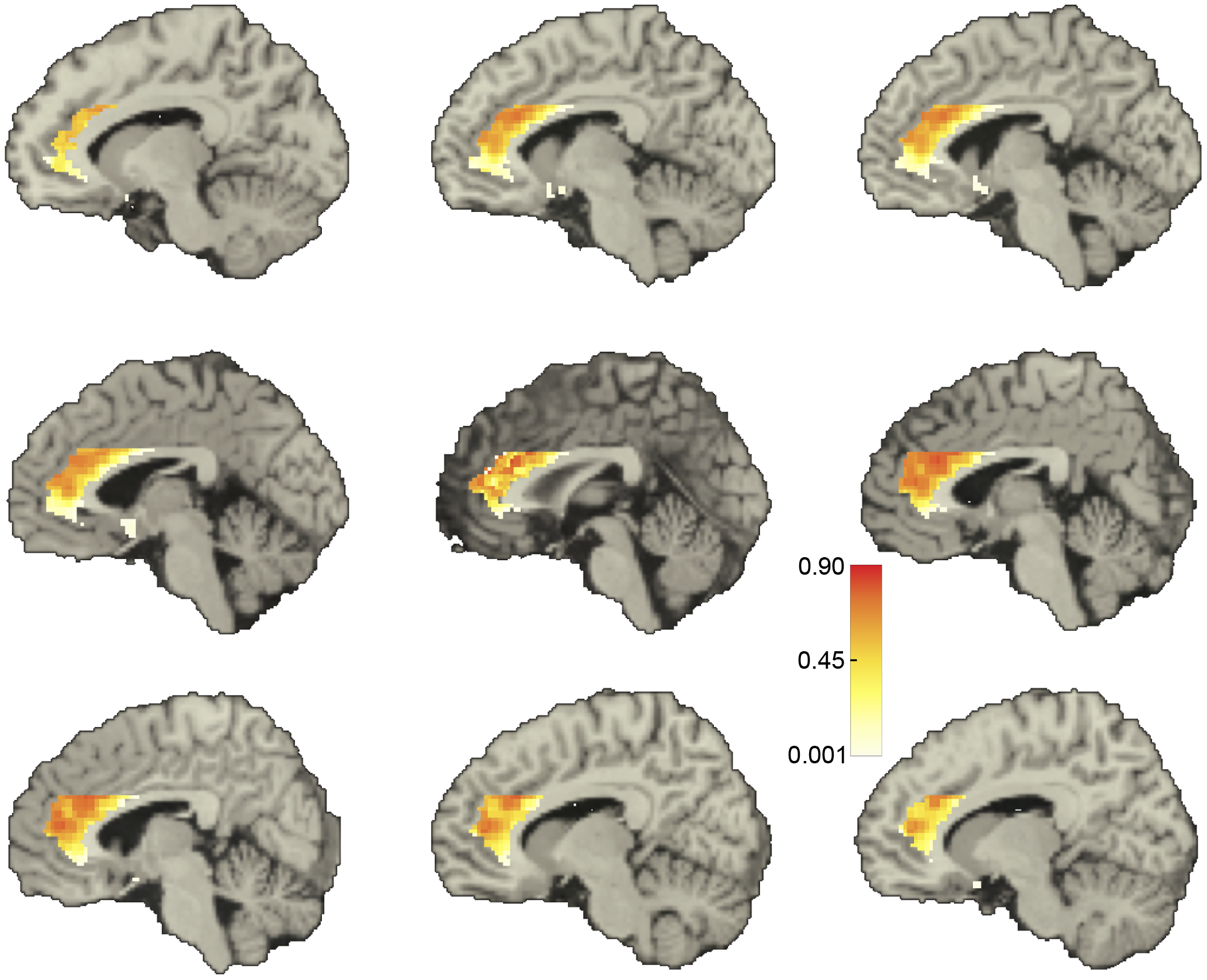

Supplement: S7 Fig — Color indicates the fraction of voxels in the ipsi-lateral amygdala with which each voxel in the ACC is significantly correlated. All significant correlations were positive. From upper left of figure: sagittal slices at x = 10, 6, 4, 2, -2, -4, -6, -10, and -12 mm. (TIF) [file pone.0130246.s007.tif]

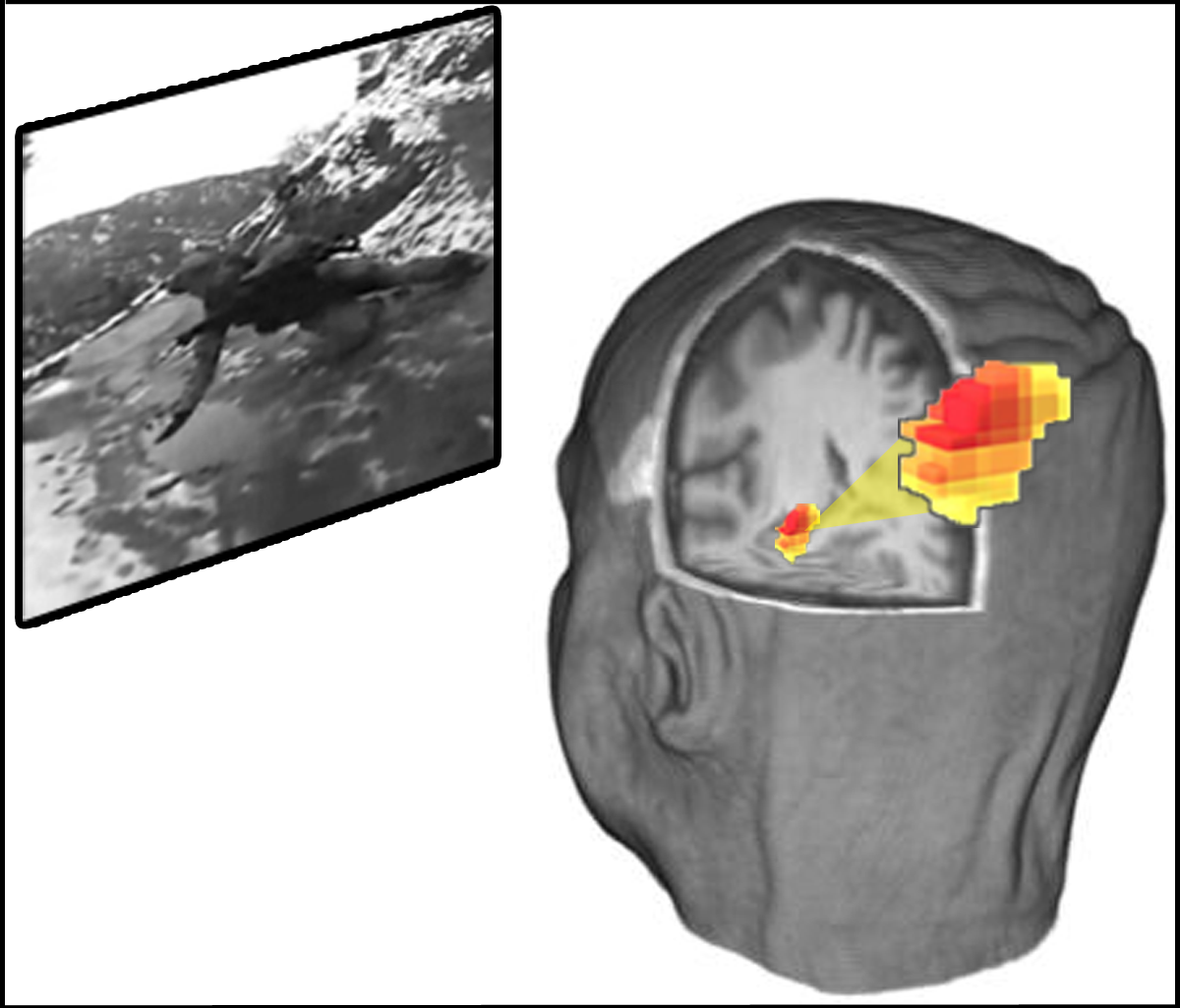

Supplement: S8 Fig — This schematic image indicates the general area of the amygdala in a cut-away view of the brain. The amygdala is colored to represent the correlation of BOLD activity with the ACC. This is done as in Figs 5 and 6. (TIF) [file pone.0130246.s008.tif]
