# Supplementary material for: Bi-Directional Tuning of Amygdala Sensitivity in Combat Veterans Investigated with fMRI
Source: PLoS One. 2015 Jun 29;10(6):e0130246. doi: 10.1371/journal.pone.0130246 (PMC4488265; doi:10.1371/journal.pone.0130246)
Supplement: S1 Text — (DOC) [file pone.0130246.s009.doc]

**S1 Text. Use of bilateral amygdala for ROI**

To preserve statistical power, we averaged across all voxels bilaterally in order to minimize the number of comparisons being made. Averaging over the entire ROI bilaterally can also be expected to minimize the effects on the signal of non-task-related artifacts (e.g., subject movement). The correlation between the signal time courses of the mean right and the mean left amygdala was 0.92, indicating a high degree of similarity between the BOLD signal time courses of the two hemispheres.
