# Supplementary material for: Bi-Directional Tuning of Amygdala Sensitivity in Combat Veterans Investigated with fMRI
Source: PLoS One. 2015 Jun 29;10(6):e0130246. doi: 10.1371/journal.pone.0130246 (PMC4488265; doi:10.1371/journal.pone.0130246)
Supplement: S2 Text — (DOC) [file pone.0130246.s010.doc]

**Text S2. Mean amygdala signal**

We chose time points that corresponded to fMRI images acquired while subjects were watching each movie and each fixation period. To allow for the hemodynamic lag, we considered all signal occurring after 2 seconds of the film had been shown and ending 2 seconds after that film’s stop as corresponding to viewing that film. In choosing what signal to include for the fixation periods, we had to take into account that the initial portions of the second fixation period would likely be influenced by lingering effects of having viewed the first film. We decided to include all signal beginning at 10 seconds after a fixation cross appeared and ending 2 seconds after the cross disappeared as corresponding to viewing the fixation cross.
