# Supplementary material for: Bi-Directional Tuning of Amygdala Sensitivity in Combat Veterans Investigated with fMRI
Source: PLoS One. 2015 Jun 29;10(6):e0130246. doi: 10.1371/journal.pone.0130246 (PMC4488265; doi:10.1371/journal.pone.0130246)
Supplement: S3 Text — (DOC) [file pone.0130246.s011.doc]

**Text S3. Similarity and differences of this method to boxcar convolution.**

The high degree of stimulus variability within each movie presents both advantages and disadvantages. The main advantage derives from what has been called the “ecological validity” of the stimulus (1). The greater the resemblance of an experimental stimulus to stimuli subjects could be expected to encounter in real-life situations, the more robust the response is likely to be. Movies provide dynamic visual and auditory stimuli and can be quite engrossing. The main disadvantage is that the stimulus presents so many overlapping events that it is quite difficult to construct an *a priori* model for what the BOLD signal time course might be. With movies, it is not even clear what constitutes an “event” to which a particular region of the brain might respond. Therefore, specification of an *a priori* model of BOLD signal responses to the movies is not clearly feasible. In order to extract a relatively model-free measurement of BOLD signal responses, we used the simple concept of averaging signal magnitude across the movie-viewing period.

To see that this method has some similarity to the method often employed when analyzing block-design fMRI studies, consider the following. In the boxcar analysis, one typically regresses a modified series of concatenated constants that vary from one another in their value. When plotted, this resembles the silhouette of a boxcar train. When fitting a flat curve of constant value to a set of data, the best fit in a least-squares sense will be the mean of that data. In this sense, our method is similar to a boxcar regression with only a single epoch.

There are both conceptual and practical differences between our method and a boxcar regression. Conceptually, the boxcar regression is a way to test how closely experimental data resemble a specific prediction of how the data should look. The summary statistic, typically the beta weight, is thus a measure of the fit of the data to the predicted model. In the limiting case of a boxcar with a single epoch, one is regressing the data on a constant. This is based on the prediction that the data vary only randomly from a constant value. The beta weight will be the mean of the data and represents the best prediction of what the constant value would be *if* the data were to come from a process that generates a constant value. The beta weight does not provide any information on whether the data actually are likely to have come from a process that generates a single constant output. In order to tell whether the data fit the predicted model, the variance of the data around the constant value becomes important.

Practically, in order to use the boxcar regression, one must have a prediction of how the BOLD signal will vary both within and between epochs. If we had chosen to use the boxcar regression to analyze our data, we would have had to predict either that BOLD signal would not vary within each movie, or we would have had to specify how it would vary. We would also have had to specify the degree to which BOLD signal would change between fixation and each movie as well as how it would change between movies.

Note also that boxcar regression (and other methods in which data is fit to a model) requires a distinction between subject-level statistics and group-level statistics. In SPM, this is contained in the distinction between the “fixed effects” of subject-level results and the “random” or “mixed effects” of the group-level results. The reason this is important is that the subject-level analysis assigns a statistical significance to each subject’s results that depends on within-subject variance in the data. (This will be the case whenever the measure of interest is how well experimental data fit an *a priori* model.) At the group level, there is additional between-subject variance. The two types of variance need to be kept separate during the group-level analysis if results are to be generalized to the general population. This distinction does not apply to our analysis because subject-level variance is not a factor. This is not to say that subject-level variance does not contain any information that could be useful, only that by choosing the mean signal as our measure of interest we are disregarding within-subject variance. Clearly, we would not be able to disregard within-subject variance if we were fitting the data to an *a priori* model.
