# Supplementary material for: Bi-Directional Tuning of Amygdala Sensitivity in Combat Veterans Investigated with fMRI
Source: PLoS One. 2015 Jun 29;10(6):e0130246. doi: 10.1371/journal.pone.0130246 (PMC4488265; doi:10.1371/journal.pone.0130246)
Supplement: S4 Text — (DOC) [file pone.0130246.s012.doc]

**Text S4. Regressors and nuisance variables.**

Our analyses followed two general models: activation-based and inter-regional correlation based (one variant of what is often called “functional connectivity”). The effects of non-task-related BOLD signal (nuisance variables) are different for each of the two models. With activation-based models, the analyses are concerned with matching BOLD signal levels to task-related variables, which in our case are the time periods during which each movie was shown. When analyzed at the group level and averaged over ROIs, the primary risk of not accounting for nuisance variables (including movement) is a lack of sensitivity due to inter-subject variance not accounted for. Because of the relatively large number of subjects in our study and the smoothing effect afforded by averaging BOLD signal over an entire ROI, we did not include nuisance regressors in our analysis of mean amygdala and ACC activation levels. Another reason we did not include motion parameter estimates was that we found that each subject’s estimated motion parameters derived with SPM were highly correlated with that subject’s global gray-matter signal (median 0.27, *p* < 0.0005 signed-rank test). Because prior studies have found that movies induce wide-spread correlations in BOLD signal throughout the brain, inclusion in our regression of motion parameters that are themselves correlated with brain-wide BOLD signal changes ran the risk of removing stimulus-related signal.

In contrast, in an analysis of inter-regional voxel-level correlations in signal, not accounting for nuisance variables (e.g., movement artifacts and non-task-related signal) can lead to systematic biases in the results. We therefore accounted for nuisance regressors in our correlation analyses as described in the Methods section.
