# Supplementary material for: Bi-Directional Tuning of Amygdala Sensitivity in Combat Veterans Investigated with fMRI
Source: PLoS One. 2015 Jun 29;10(6):e0130246. doi: 10.1371/journal.pone.0130246 (PMC4488265; doi:10.1371/journal.pone.0130246)
Supplement: S5 Text — (DOC) [file pone.0130246.s013.doc]

**Text S5. Correlating CAPS scores with voxel-level inter-ROI BOLD-signal correlations**

In this analysis, each of the 10,000 iterations resulted in a single correlation matrix rather than one correlation matrix for each participant as had been the case for the amy_m_::acc_n_ permutations. This is because each iteration calculated a correlation across subjects (that between subjects’ amy_m_::acc_n_ and subjects’ randomly assigned CAPS score) rather than within each subject (that between each subject’s amygdala and ACC voxels). This allowed us to use the 10,000 randomly determined correlation matrices directly to calculate z-scores for the actual data’s empirically observed correlation matrix in the standard fashion (dividing the difference between the empirically observed correlation matrix and the mean of the 10,000 random correlation matrices by the standard deviation of the 10,000 random correlation matrices). Again, we used ± 5.39 as the criterion z-score for significance.
