# Supplementary material for: Bi-Directional Tuning of Amygdala Sensitivity in Combat Veterans Investigated with fMRI
Source: PLoS One. 2015 Jun 29;10(6):e0130246. doi: 10.1371/journal.pone.0130246 (PMC4488265; doi:10.1371/journal.pone.0130246)
Supplement: S6 Text — (DOC) [file pone.0130246.s014.doc]

**Text S6. Analysis with Categorical Division of Subjects**

We divided subjects into two groups based on CAPS scores. We considered subjects with CAPS < 45 (n = 18) to be without PTSD, and those with CAPS scores ≥ 45 (n = 32) to have PTSD. Mean amygdala BOLD signal during the civilian movie was significantly higher in subjects with PTSD than those without (*t*(48) = 4.3, *p* = 0.044). Mean amygdala BOLD signal during the combat movie was significantly lower in subjects with PTSD (*t*(48) = 4.9, *p* = 0.031). Mean amygdala BOLD signal during the combat movie minus that during the civilian movie was significantly lower in subjects with PTSD (*t*(48) = 8.9, *p* = 0.0044).
