# Supplementary material for: Bi-Directional Tuning of Amygdala Sensitivity in Combat Veterans Investigated with fMRI
Source: PLoS One. 2015 Jun 29;10(6):e0130246. doi: 10.1371/journal.pone.0130246 (PMC4488265; doi:10.1371/journal.pone.0130246)
Supplement: S1 Table — (DOC) [file pone.0130246.s019.doc]

**Table S1. Comparison of Results For All Subjects to Male-Only Subjects**

We checked to see whether excluding the two female subjects from the analysis would alter the results in any appreciable way. We found that they did not.

| Variable correlated with CAPS score | All Subjects  (n = 50) | Male Subjects only  (n = 48) |
| --- | --- | --- |
| Mean amygdala signal during less-arousing movie | *r* = 0.40, *p* = 0.0041 | *r* = 0.40, *p* = 0.0046 |
| Mean amygdala signal during more-arousing movie | *r* = -0.29, *p* = 0.039 | *r* = -0.29, *p* = 0.045 |
| Difference in mean amygdala signal | *r* = -0.46, *p* = 0.00089 | *r* = -0.45, *p* = 0.0012 |
| Correlation between amygdala ROI and ACC ROI | *r* = -0.34, *p* = 0.015 | *r* = -0.37, *p* = 0.001 |
