# Supplementary material for: Bi-Directional Tuning of Amygdala Sensitivity in Combat Veterans Investigated with fMRI
Source: PLoS One. 2015 Jun 29;10(6):e0130246. doi: 10.1371/journal.pone.0130246 (PMC4488265; doi:10.1371/journal.pone.0130246)
Supplement: S2 Table — (DOC) [file pone.0130246.s020.doc]

**Table S2. Inclusion of additional predictive variables.**

Our *a priori* hypothesis was that PTSD symptoms (as measured by CAPS scores) would affect fMRI BOLD signal. We therefore calculated a single-predictor regression model for each of the four fMRI variables of interest: mean amygdala signal during the less-arousing movie, mean amygdala signal during the more-arousing movie, difference between mean amygdala signal during the more-arousing and less-arousing movies, and correlation between the time course of the amygdala signal and that of the ACC signal. These are the models reported in the main paper.

After running these single-regression models, we wanted to see whether the addition of some combination of a number of other predictors might better account for the fMRI variables. Our motivation for doing this was *post hoc* in nature. That is, we were not attempting to find the model that was in some specified sense “best” overall. Rather, we wanted to look for evidence that we may have missed an important predictive variable whose inclusion might remove the effect of CAPS scores. To this end, we performed an all-models multiple regression analysis using the variables listed in Table 1 in the main text: CAPS, age, number of deployments, MDD, AUD, SUD, medication use, history of mTBI, and months since mTBI or deployment. We also included a variable for combat experience, one for severity of stressors prior to combat, and one for order of movie presentation.

We therefore constructed the list of all possible combinations of all variables. We excluded all combinations that were merely re-orderings of elements. The length of such non-re-ordering combinations is given by 2*^n^* -1 (excluding the null set). Because we had 12 variables, there were 4,095 models, one for each combination of elements. For each fMRI (dependent) variable, we calculated the Akaike Information Criterion (AIC) value for each model and kept the model with the best (lowest) AIC value. Even though there was no requirement that the best model had to include CAPS scores, each model chosen in this manner included CAPS score as a significant predictor variable. In fact, CAPS score in each case was the predictor variable with the largest (or most negative) *t*-value. The predictive variables for each best-fit model are listed in Table S2 below along with each variable’s *t*-value and *r*-value. We also included the value of each model’s *r*-square.

Table S2

|  | Variables included in (AIC) best-fit model | *t*-value | *p*-value | *r*-squared for model |
| --- | --- | --- | --- | --- |
| Mean amygdala signal during less-arousing movie | CAPS  Order of movies | 3.2  -1.98 | 0.0026  0.053 | 0.22 |
| Mean amygdala signal during more-arousing movie | CAPS  Age  Medication use | -2.22  -2.09  1.92 | 0.031  0.042  0.061 | 0.24 |
| Difference in mean amygdala signal (more- minus less-arousing movie) | CAPS  Order of movies  Medication use | -4.42  2.39  2.05 | 0.000060  0.021  0.047 | 0.36 |
| Correlation between amygdala and ACC BOLD signal | CAPS  Age  Order of movies  No mTBI  MDD  mTBI (possible) | -3.03  -2.99  1.98  -1.96  -1.86  -1.29 | 0.0042  0.0047  0.055  0.056  0.070  0.20 | 0.40 |
