# Supplementary material for: Bi-Directional Tuning of Amygdala Sensitivity in Combat Veterans Investigated with fMRI
Source: PLoS One. 2015 Jun 29;10(6):e0130246. doi: 10.1371/journal.pone.0130246 (PMC4488265; doi:10.1371/journal.pone.0130246)
Supplement: S3 Table — (DOC) [file pone.0130246.s021.doc]

**Table S3. Analysis of First 27 and Last 23 Subjects Separately**

We monitored the gaze of the final 23 participants with an Avotec RE-5701 Eye Tracker. In order to see whether the fMRI results of the subjects whose gaze was not monitored were similar to the results of the subjects whose gaze was monitored, we analyzed each subset of subjects separately. The results where similar in each subset to those of the entire group. When analyzed separately these often, although not always, reached statistical significance. Because the direction of the results is the same in both groups, we believe it most likely that the reduced sample size is responsible for the lack of statistical significance in those cases in which it is found.

Table S2

| Variable correlated with CAPS score | First 27 subjects | Last 23 subjects |
| --- | --- | --- |
| Mean amygdala signal during less-arousing movie | r = 0.50, p = 0.0069 | r = 0.26, p = 0.27 |
| Mean amygdala signal during more-arousing movie | r = -0.3, p = 0.11 | r = -0.42, p = 0.046 |
| Difference in mean amygdala signal | r = -0.49, p = 0.0094 | r = -0.44, p = 0.035 |
| Correlation between amygdala ROI and ACC ROI | r = -0.21, p = 0.29 | r = -0.55, p = 0.0058 |
