# Supplementary material for: Bi-Directional Tuning of Amygdala Sensitivity in Combat Veterans Investigated with fMRI
Source: PLoS One. 2015 Jun 29;10(6):e0130246. doi: 10.1371/journal.pone.0130246 (PMC4488265; doi:10.1371/journal.pone.0130246)
Supplement: S4 Table — (DOC) [file pone.0130246.s022.doc]

**Table S4. CAPS Sub-scales Correlation with SCRs**

|  | Avoidance | Arousal | Re-experiencing |
| --- | --- | --- | --- |
| SCRs during civilian movie | *ρ* = -0.21, *p* = 0.20 | *ρ* = -0.20, *p* = 0.20 | *ρ* = -0.28, *p* = 0.07 |
| SCRs during combat movie | *ρ* = 0.01, *p* = 0.96 | *ρ* = -0.05, *p* = 0.77 | *ρ* = -0.20, *p* = 0.21 |
| SCRs during combat – SCRs during civilian movie | *r* = 0.22, *p* = 0.16 | *r* = 0.12, *p* = 0.42 | *r* = 0.11, *p* = 0.48 |

Correlation coefficients for CAPS sub-scores and SCRs for the 43 subjects with SCR data. Correlations are Pearson’s correlation ( *r* ) for data that are normally distributed and Spearman’s correlation ( *ρ* ) otherwise.
